# Complement lectin pathway activation is associated with COVID-19 disease severity, independent of *MBL2* genotype subgroups

**DOI:** 10.3389/fimmu.2023.1162171

**Published:** 2023-03-27

**Authors:** Lisa Hurler, Ágnes Szilágyi, Federica Mescia, Laura Bergamaschi, Blanka Mező, György Sinkovits, Marienn Réti, Veronika Müller, Zsolt Iványi, János Gál, László Gopcsa, Péter Reményi, Beáta Szathmáry, Botond Lakatos, János Szlávik, Ilona Bobek, Zita Z. Prohászka, Zsolt Förhécz, Dorottya Csuka, Erika Kajdácsi, László Cervenak, Petra Kiszel, Tamás Masszi, István Vályi-Nagy, Reinhard Würzner, Cambridge Institute of Therapeutic Immunology and Infectious Disease-National Institute of Health Research (CITIID-NIHR) COVID BioResource Collaboration, Stephen Baker, Paul A. Lyons, Erik J. M. Toonen, Zoltán Prohászka

**Affiliations:** ^1^ Department of Internal Medicine and Haematology, Semmelweis University, Budapest, Hungary; ^2^ Cambridge Institute of Therapeutic Immunology and Infectious Disease, Jeffrey Cheah Biomedical Centre, University of Cambridge, Cambridge, United Kingdom; ^3^ Department of Medicine, University of Cambridge, Addenbrooke’s Hospital, Cambridge, United Kingdom; ^4^ Research Group for Immunology and Haematology, Semmelweis University - Eötvös Loránd Research Network (Office for Supported Research Groups), Budapest, Hungary; ^5^ Department of Haematology and Stem Cell Transplantation, Central Hospital of Southern Pest - Institute of Haematology and Infectious Diseases, Budapest, Hungary; ^6^ Department of Pulmonology, Semmelweis University, Budapest, Hungary; ^7^ Department of Anaesthesiology and Intensive Therapy, Semmelweis University, Budapest, Hungary; ^8^ Department of Infectology, Central Hospital of Southern Pest - Institute of Haematology and Infectious Diseases, Budapest, Hungary; ^9^ Department of Anaesthesiology and Intensive Therapy, Central Hospital of Southern Pest - Institute of Haematology and Infectious Diseases, Budapest, Hungary; ^10^ Institute of Hygiene and Medical Microbiology, Medical University of Innsbruck, Innsbruck, Austria; ^11^ Research and Development Department, Hycult Biotech, Uden, Netherlands

**Keywords:** mannose binding lectin (MBL), MBL2 genotypes, COVID-19, severe acute respiratory coronavirus 2 (SARS-CoV-2), lectin pathway activation, lectin pathway of complement

## Abstract

**Introduction:**

While complement is a contributor to disease severity in severe acute respiratory syndrome coronavirus 2 (SARS-CoV-2) infections, all three complement pathways might be activated by the virus. Lectin pathway activation occurs through different pattern recognition molecules, including mannan binding lectin (MBL), a protein shown to interact with SARS-CoV-2 proteins. However, the exact role of lectin pathway activation and its key pattern recognition molecule MBL in COVID-19 is still not fully understood.

**Methods:**

We therefore investigated activation of the lectin pathway in two independent cohorts of SARS-CoV-2 infected patients, while also analysing MBL protein levels and potential effects of the six major single nucleotide polymorphisms (SNPs) found in the MBL2 gene on COVID-19 severity and outcome.

**Results:**

We show that the lectin pathway is activated in acute COVID-19, indicated by the correlation between complement activation product levels of the MASP-1/C1-INH complex (p=0.0011) and C4d (p<0.0001) and COVID-19 severity. Despite this, genetic variations in MBL2 are not associated with susceptibility to SARS-CoV-2 infection or disease outcomes such as mortality and the development of Long COVID.

**Conclusion:**

In conclusion, activation of the MBL-LP only plays a minor role in COVID-19 pathogenesis, since no clinically meaningful, consistent associations with disease outcomes were noted.

## 1 Introduction

After the first severe acute respiratory syndrome coronavirus 2 (SARS-CoV-2) infection was reported in December 2019 in Wuhan, China, the virus has spread globally in the last three years. As of February 1, 2023, more than 753 million COVID-19 cases have been reported worldwide, resulting in a total of more than 6.8 million deaths ((1), Weekly epidemiological update on COVID-19 –1 February 2023).

A major primary defence mechanism of the innate immune response against viruses is the complement system, a proteolysis-based activation cascade consisting of more than 40 plasma proteins. Upon recognition of viruses or viral particles, this system has four major functions: lysis of infected cells or enveloped viruses *via* formation of the membrane attack complex (MAC complex; also referred to as TCC or C5b-9) (2, 3), direct opsonization of viral particles (4), solubilisation of antibody-virus complexes (5), and activation of inflammation in the host (6). The complement system can be activated *via* three different pathways, the classical (CP), the lectin (LP), and the alternative pathway (AP), all leading to the formation of a C3 convertase, downstream activation of the complement cascade, and ultimately to the formation of the MAC complex (7).

Although the main function of the complement system in viral infections is to protect the host from invading viruses, complement over-activation also seems to play a role in the pathogenesis of COVID-19. Indeed, several studies reported that complement activation was associated with higher disease severity, ICU admittance and increased mortality (8–10). In addition, clinical trials with complement inhibitors targeting either C5 or C3 in the treatment of severe COVID-19 cases are ongoing (11). Despite it is now commonly accepted that complement has a role in the onset and disease course of COVID-19, it is not precisely understood how or *via* which activation pathway complement contributes to the pathogenesis. Already very early after SARS-CoV-2 started spreading worldwide, it was suggested that activation of the lectin pathway was responsible for (at least) some of the complement-mediated effects in COVID-19 (12–14).

Six pattern recognition molecules (PRMs) are able to activate the lectin pathway in humans, namely the three collectins mannan-binding lectin (MBL), collectin -10 and -11, as well as ficolins -1, -2, and -3 (15–17). These PRMs are bound to the MBL-associated serine proteases (MASPs) 1-3. MASP-1 and MASP-2 are present in a zymogen form. Upon binding to carbohydrate structures or acetyl group moieties, as found on the surface of pathogens like viruses, fungi or bacteria, zymogen MASP-1 can auto activate, cleave MASP-2 and subsequently MASP-2 cleaves complement components C4 and C2, followed by formation of the classical C3 convertase (C4b2b) and downstream complement activation (18). Although MASP-1 is not able to cleave C4 (19), it initiates LP-triggered C3 convertase formation by activating MASP-2 *via* proteolytic cleavage (20, 21). Early evidence suggesting that lectin pathway activation contributes to disease course and/or complications in COVID-19 was provided by Eriksson and co-workers (12). They showed that high MBL levels, and in consequence activation of the lectin pathway, is associated with thromboembolic complications in critically ill COVID-19 patients. Activated platelets and fibrin, generated during thrombotic events, may activate MASP-1 and MASP-2 during blood clotting, and thereby connect thromboinflammation and lectin pathway activation (22). While deposition of MASP-2, C4d, C5b-9 and MBL was shown in lung tissue from COVID-19 patients, increased complement activation product levels were observed in patient sera from SARS-CoV-2 infected individuals (13, 14, 23). Although only a small number of patients was investigated in those early studies pointing towards lectin pathway involvement in the pathophysiology of COVID-19, Rambaldi et al. utilized the lectin pathway inhibitor Narsoplimab for COVID-19 treatment, and could show recovery after antibody administration in a total of six treated patients (24). Ali et al. showed that MBL, along with other recognition molecules of the LP of complement, can bind to spike (S) and nucleocapsid (N) proteins of SARS-CoV-2 and demonstrated an LP-mediated deposition of C3b and C4b molecules on SARS-CoV-2 proteins *in vitro* (25). In contrast, Stravalaci and colleagues could only verify binding of MBL to SARS-CoV-2 spike, while other lectin pathway PRMs did not bind to the viral proteins (26). Although these findings indicate activation of the lectin pathway, the exact mechanisms underlying its role in the pathophysiology in COVID-19 are still not fully understood. Particularly, the influence of *MBL2* genetic variations have never been evaluated on MBL binding to SARS-CoV-2 components.

Activatability of the lectin pathway is highly dependent on the level of its key pattern-recognition molecule, mannose-binding lectin (reviewed in (27)). The serum functional MBL concentration shows high variability, which is mainly genetically determined: an interplay between promoter and structural gene polymorphisms influences basal serum levels ranging from undetectable to as high as 10 µg/mL. The first exon of the *MBL2* gene, encoding the MBL protein, may contain three missense polymorphisms at codons 54 (Gly54Asp; rs1800450), 57 (Gly57Glu; rs1800451) or 52 (Arg52Cys; rs5030737) (28–30) (summarized in **Supplementary Figure 1**). The variant alleles are termed B, C or D, respectively, and any of these variants on the chromosome is referred to as the 0 allele, while the wildtype allele is named A. Occurrence of either substitution causes disturbance in the structure of the collagen-like domain and diminished stability of the higher-order forms, and thus results in markedly decreased functional MBL levels and reduced activation of the lectin pathway. In addition, three common polymorphisms of the promoter/5’-UTR region (-550C/G: L/H, rs11003125; -221G/C: Y/X, rs7096206; +4C/T: P/Q, rs7095891) may also affect MBL serum levels, the highest effect is attributed to the Y/X variation. These polymorphisms are in strong linkage disequilibrium, leading to the formation of seven common haplotypes, that display high (HYPA, LYQA), intermediate (LYPA), low (LXPA) or null (LYPB, HYPD, LYQC haplotypes) biological activity (reviewed in detail in (27, 31, 32). If only variants with greater importance are considered, *MBL2* haplotypes are often referred to by their short names in the literature, namely YA for HYPA, LYQA, LYPA; XA for LXPA and 0 for LYPB, HYPD, LYQC. Functional MBL deficiency, resulting from the combination of low/null haplotypes (XA/0, 0/0), is one of the most common immunodeficiencies associated with an increased susceptibility to certain extracellular pathogens presumably in childhood and in immunocompromised conditions (33). In general, around 10-30% of the population are MBL-deficient, depending on the definition of deficiency either by antigenic MBL levels (<500 ng/mL (34)), or MBL function (<0.2 U/μL C4b deposition (35)).

However, MBL acts as a double-edged sword; high MBL level and lectin pathway activity causing excessive complement activation has also been associated with different pathological states like inflammatory diseases, transplant rejection, and diabetic nephropathy (36). In COVID-19, the role of MBL has not been fully explored. Several studies showed lectin pathway activation, high tissue expression of MBL in lungs of patients deceased from severe SARS-CoV-2 infection, as well as a potential link between disease severity or outcome and *MBL2* polymorphisms (26, 37, 38), while others could not fully replicate those findings (39).

**Figure 1:**
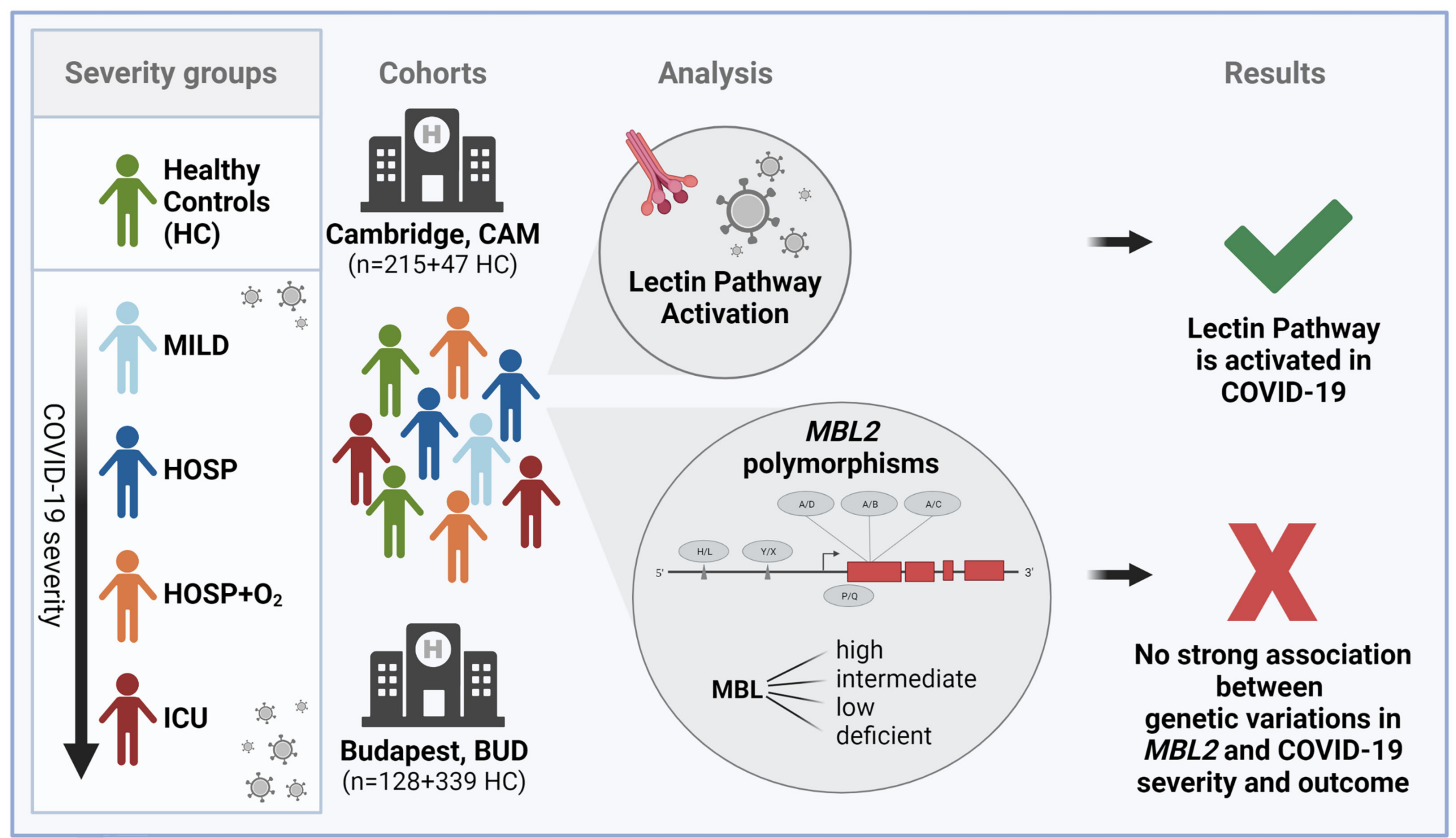
Graphical summary of the study. Figure created with BioRender.com.

We therefore aimed to investigate the role of lectin pathway activation in COVID-19 in two independent patient cohorts (9, 40), while also looking at the six most common polymorphisms found in the *MBL2* gene (as summarized in **Figure 1** and **Supplementary Figure 1**).

Our results suggest that the lectin pathway is activated in COVID-19 patients, with higher levels of the specific LP activation marker MASP-1/C1-INH complex, and the joint LP/CP marker C4d in more severe cases. Nonetheless, the observed lectin pathway activation seems to be independent of the *MBL2* genetic background of the individuals.

## 2 Materials and methods

### 2.1 Patient cohorts

Two patient cohorts were included in this study, in addition to one cohort consisting of healthy volunteers. One-hundred and fifty-nine COVID-19 patients at or soon after admission to Addenbrooke’s or Royal Papworth’s hospitals (Cambridge, UK), and 56 SARS-CoV-2 positive healthcare workers without symptoms, or with mild symptoms (identified in routine screening) were enrolled to the first cohort between 31st of March and 20th of July, 2020 (cohort described in detail in (40)). So altogether, 215 COVID-19 patients, next to 47 healthy controls (HC), were enrolled in Cambridge (CAM). Ethical approval was obtained from the East of England – Cambridge Central Research Ethics Committee (“NIHRBioResource” REC ref 17/EE/0025, and “Genetic variation AND Altered Leucocyte Function in health and disease – GANDALF” REC ref 08/H0308/176). The final analysis included only patients with available data on severity, *MBL2* genotype as well as biomarker measurements.

The second patient cohort (BUD) was recruited in Budapest, Hungary, between April and July 2020 (BUD cohort (9)). Briefly, 102 adult patients hospitalized for COVID-19 disease and 26 outpatients with evidence of past SARS-CoV-2 infection (CONV) were enrolled based on a study protocol approved by the Hungarian Ethical Review Agency (ETT-TUKEB; Bo IV/4403-2/2020/EKU).

For both studies, written informed consent was obtained from the patients or their closest relative, and the Declaration of Helsinki was followed. Sampling was done at or soon after infection or symptom onset, and only one sample per patient was included in the presented results. Of note, the 26 individuals of the Budapest cohort not requiring hospitalization (MILD/CONV, BUD) were sampled in the convalescent phase after their SARS-CoV-2 infection. Because of that, measurements of complement markers were excluded from biomarker analysis, since sampling was not done in the acute stage of COVID-19. However, the individuals were included in genetic analysis.

For genetic analysis, a third cohort of 339 historical healthy Hungarians (Healthy controls (BUD)) was included in the analysis, while ethical approval was obtained by the Scientific and Research Ethics Committee of the Medical Research Council (ETT TUKEB) in Budapest, Hungary (8361-1/2011-EKU).

Blood samples were drawn and directly transferred to the processing laboratory, where cells and supernatant – serum and EDTA-anticoagulated plasma – were separated by centrifugation. Serum and plasma aliquots were immediately frozen and stored at -80°C until measurements or further processing.

### 2.2 Clinical data collection

Clinical and laboratory data were collected from electronic patient charts and hospital records, and were validated by a physician (including COVID-19 related death/mortality as an outcome in cases). Development of Long COVID, defined as persisting symptoms 6-12 months after the SARS-CoV-2 infection, was investigated as a second outcome in the Cambridge cohort, using *ad hoc* validated questionnaires. Therefore, the questionnaire for assessment of long-term outcomes following COVID-19 was based on a published tool developed by Cambridge University Hospitals for assessing rehabilitation need in patients who had prolonged ICU stays following COVID-19 infection (41). The modified tool, assessing a range of long-term self-reported outcomes, was administered at approximately 3-5 months and 9-10 months post symptom onset to patients. Participants were asked to report only on symptoms arising, or worsening in severity, following SARS-CoV-2 exposure and scores across seven symptom categories (fatigue, dyspnea, cough, pain, cognition and memory, new neurology, and muscle weakness) were used to classify individuals into “Long COVID” symptom groups.

### 2.3 DNA extraction and analysis of the *MBL2* gene

#### 2.3.1 Budapest (BUD)

DNA was extracted from peripheral blood mononuclear cells (PBMCs) collected in EDTA plasma, according to the standard salting out procedure described by Miller et al. (42).

Four SNPs (Y/X, A/B, A/C, A/D) were analysed using real-time polymerase chain reaction (PCR) with four different TaqMan^®^ SNP Genotyping Assays (Life Technologies): C:27858274_10 for identification of the allele Y/X (rs7096206), C:_2336609_20 for A/B (rs1800450), C:_2336608_20 for A/C (rs1800451), and C:_2336610_10 for A/D (rs5030737). The reactions were performed separately using the Rotor-Gene Q instrument (QIAGEN, Hilden, Germany) according to the manufacturer’s instructions.

Genotype of two promoter SNPs (L/H-rs11003125 and P/Q-rs7095891) were determined by Sanger-sequencing (with two forward primers: 5’-TCAAAGGGAAACTTGGAGGCTT -3’ for L/H and 5’-GCACAGATGAACCCCTCCTTAG-3’ for P/Q) following PCR amplification (with the following primer pairs: F: 5’-AGTCAACTACCTCACCTCACC-3’ and R: 5’-CTGGGCTGGCAAGACAACTA-3’). Following purification of PCR products with Exonuclease I and FastAP Thermosensitive Alkaline Phosphatase (Thermo Scientific, Waltham, MA, US), sequencing was performed with BigDye Terminator v3.1 Cycles Sequencing Kit (Life Technologies, Carlsbad, CA, US) according to the manufacturer’s instructions. After sodium acetate/ethanol purification, sequencing products were separated with an Applied Biosystems 3130xl Genetic Analyzer (Life Technologies, Carlsbad, CA, US).

#### 2.3.2 Cambridge (CAM)

Genotyping was performed as previously described (43). Briefly, genomic DNA was extracted from peripheral blood mononuclear cells using the Qiagen All Prep kit according to the manufacturer’s instructions. Genotyping was performed using the Affymetrix UK Biobank Axiom Array by Cambridge Genomic Services (Cambridge, UK). Genotype calling was performed using the Affymetrix Powertools software and after genotype calling *MBL2*-related single nucleotide polymorphisms (SNPs) were extracted from the acquired data using plink and haplotypes were determined based on the haplotype frequency in the normal human population.

#### 2.3.3 Long MBL haplotypes

Long MBL haplotypes were derived using the PHASE (v2.1) Program (44, 45). Each but one haplotype combination could be predicted with a probability ≥99%, only the LYPA/HYPD and HYPA/LYPD combinations could not be distinguished unequivocally, hence they are combined in the analysis as one single group (HYPA/LYPD).

### 2.4 Determination of complement and laboratory parameters

#### 2.4.1 Budapest (BUD)

C-reactive protein (CRP) and interleukin-6 (IL-6) levels, as well as complete blood count parameters were measured at clinical laboratories of the two hospitals where the patients were treated, as described earlier (9). Required data was extracted from hospital records. Concentrations of the terminal pathway activation marker sC5b/9/TCC (A020, Quidel), anaphylatoxin C3a (A031, Quidel), activity of the MBL-lectin pathway LP% (COMPLMP320RUO, SVAR Life science), MBL (HK323, Hycult Biotech; determination of MBL concentrations in the BUD cohort in healthy controls only), C4d (A008, Quidel) and MASP-1/C1-INH complex (HK3001, Hycult Biotech, (46)) were determined using commercially available assays according to manufacturer’s instructions, as partially described elsewhere (9). Concentrations of C1-inhibitor protein were determined by radial immunodiffusion using a specific polyclonal antibody (47).

#### 2.4.2 Cambridge (CAM)

Determination of general laboratory parameters and complement C3a (HK354, Hycult Biotech), C3c (HK368, Hycult Biotech) and C5b-9/TCC levels (HK328, Hycult Biotech) has already been described before (40). Additional measurements of MBL (HK323), C1-INH (HK396) and MASP-1/C1-INH complex levels (HK3001) were done in EDTA plasma samples using commercially available ELISA kits (Hycult Biotech) according to the manufacturer’s instructions.

### 2.5 Binding of MBL from human sera to SARS-CoV-2 spike protein

Recombinant SARS-CoV-2 spike protein (SPN-C52H9, AcroBiosystems) was coated on 96-well plates overnight at 4°C in a 2-fold serial dilution starting from 50 pmol/mL (equals 5 pmol/well) in phosphate-buffered saline (PBS). As a control for MBL binding, some wells were coated with 10 μg/mL mannan (M7504, Sigma-Aldrich) in PBS.

After coating, wells were blocked with 200 μL of 2% bovine serum albumin (BSA) in TBST-Ca^2+^ buffer (Tris-HCl buffer containing 150 mM NaCl, 2 mM CaCl_2_ and 0.1% Tween 20, pH 7.5) for 2 h at 37°C. Plates were then washed three times with TBST-Ca^2+^ and incubated with 100 μL of either 10% serum in TBST-Ca^2+^, 1000 ng/mL recombinant MBL (rMBL, positive control; gift by S. Thiel et al., produced as described in (48)) or 5% BSA in TBST-Ca^2+^ (negative control) for 1 h at 37°C. Then, plates were washed three times again, before incubation was performed for 1 h at 37°C with a monoclonal anti MBL antibody (HYB131-01, SSI) in a concentration of 2 μg/mL in TBST-Ca^2+^. After three washes, plates were incubated with 1:4000 diluted HRP-labelled goat anti-mouse antibody (1010-05, Southern Biotech) for 1 h at 37°C. Afterwards, plates were incubated with TMB substrate for 5 min at room temperature, before the reaction was stopped by addition of oxalic acid, and the absorbance (OD values) was measured at 450 nm on a microplate reader. OD values of uncoated wells were subtracted from wells coated with spike protein, and binding was evaluated based on the measured absorbance.

### 2.6 Statistical analysis

Categorical data are reported as numbers with frequencies (%) and were analysed by the Fisher’s exact test with odds ratios (OR) and 95% confidence intervals. As most of the continuous variables showed skewed distributions, data are presented as medians and interquartile ranges (IQR), and non-parametric statistical tests were used (Spearman rank correlation test, Mann–Whitney test) for two-, and Kruskal-Wallis test (with Dunn’s post-test) for multiple independent groups. The level of significance was corrected by the Benjamini-Hochberg procedure in the case of multiple comparisons to maintain a false discovery rate of 5%. Statistical analysis were performed with the GraphPad Prism 9 software (GraphPad Softwares Inc., La Jolla, CA, USA) or by Statistica 13.5 (TIBCO Softwares Inc., Palo Alto, CA USA).

## 3 Results

### 3.1 Cohort overview

Baseline characteristics of the two COVID-19 cohorts as well as of the healthy controls are listed in **Table 1**, while data regarding general complement activation (C3 activation, C5b-9/TCC) in the two cohorts has been published before (9, 40). The Hungarian COVID-19 cohort (BUD) consisted of 128 patients, among whom 26 individuals (20.3%) had only mild disease and were not admitted to the hospital (sampled in convalescence; CONV) and 27 patients (21.1%) were hospitalized but did not require additional O_2_ or ventilation (moderate group (MOD)). A total of 33 hospitalized patients (25.8%) did require O_2_ and 42 individuals (32.8%) ended up in the ICU (severe group (SEV)).

**Table 1:** Baseline characteristics of healthy controls and the two COVID-19 cohorts enrolled in Cambridge (CAM) and Budapest (BUD).

Variables	COVID Cambridge (CAM)	COVID Budapest (BUD)	Healthy controls (CAM)	Healthy controls (BUD)
**total, n**	215	128	47	339
**Male sex, n (%)**	118 (54.9)	71 (55.5)	26 (55.3)	154 (45.4)
**Mean age ± SD**	52.9 ± 17.8	60.5 ± 16.5	42.3 ± 15.0	42.1 ± 12.8
**Delay between first symptom and sampling, days median (IQR)**	11 (6-31)	9 (5-20)	–	–
MODERATE cases	n (% of total cases)	n (% of total cases)		
**not requiring hospitalization (MILD/CONV^§^)**	56 (26.0)	26 (20.3)^§^	–	–
**hospitalized, but not requiring O_2_ or ventilation (HOSP)**	47 (21.9)	27 (21.1)	–	–
SEVERE cases				
**hospitalized, requiring O_2_ (HOSP+O_2_)**	40 (18.6)	33 (25.8)	–	–
**Intensive care unit (ICU)**	72 (33.5)	42 (32.8)	–	–
Laboratory findings, median (IQR)				
**C-reactive protein [ref <10 mg/L]**	25.5 (3.1-122.3)	29.4 (3.7–107.6)	1.8 (1.2-2.4)	2.1 (1.1-4.2)
**Interleukin 6 [ref 2–4.4 pg/mL]**	2.3 (0.5-11.2)	24.2 (7.1–67.9)	0.2 (0.2-0.2)	NA
**TCC/C5b-9 [ref <1000 mAU/mL^¶^]**	3804 (2913-6144)	–	2104 (1753-2527)	–
**sC5b-9 [ref 110-252 ng/mL]**	–	265 (185-380)	–	198 (148-298)
Disease outcome				
Long COVID*, n (%)	32 (53.3)	NA	–	–
**COVID-19 related death/mortality, n (%)**	19 (8.8)	25 (19.5)	–	–

^§^ The 26 patients of the MILD group in the Budapest COVID-19 cohort (BUD) were sampled in the convalescent phase of the patients, instead of the acute phase. Because of that, biomarker measurements were excluded from the analysis in merged analysis and correlations between complement and laboratory markers, while the individuals were not excluded in genetic investigations. If measurements of those 26 patients are shown, the group is termed CONV.

^¶^Reference range provided by the manufacturer, based on determinations using a preliminary version of the assay.

* The total amount of patients who were administered the questionnaire regarding persisting symptoms (Long COVID) was 60, while no questionnaires were performed/sent out in the Budapest cohort. NA, data not available.

**Figure 2:**
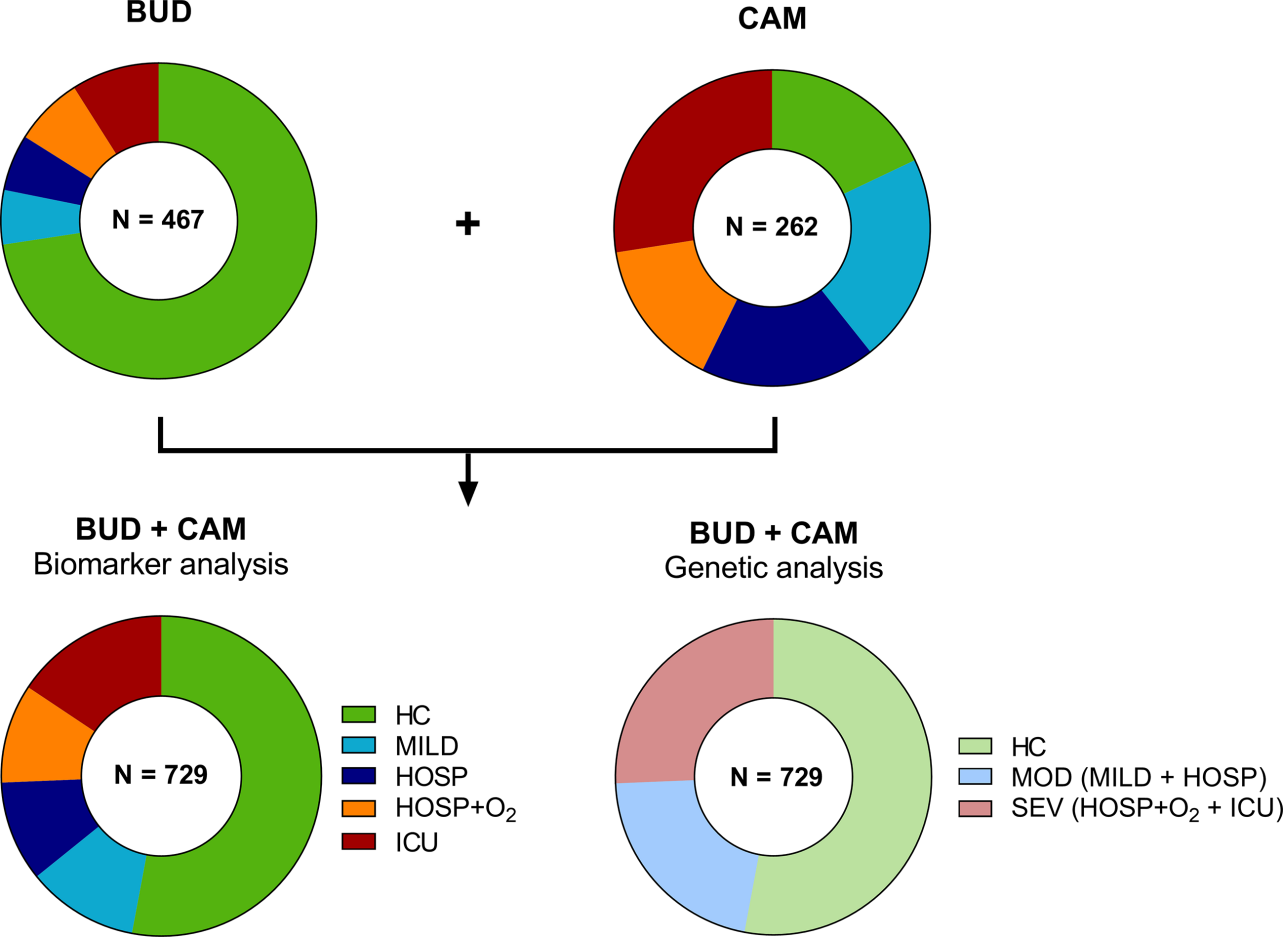
Distribution of cases and controls of the BUD and CAM cohorts for biomarker and genetic analysis. A total of 467 individuals were enrolled in Budapest (BUD), while 262 individuals were enrolled in Cambridge, UK (CAM). Individuals were stratified according to five severity groups for biomarker analysis (HC, MILD, HOSP, HOSP+O_2_, ICU), while genetic analysis were performed in only three severity groups (HC, MOD, SEV), merging MILD and HOSP into the moderate (MOD), and HOSP+O_2_ and ICU into the severe (SEV) group. BUD, Budapest/Hungarian cohort; CAM, Cambridge cohort; N, number; HC, Healthy controls; MILD, patients not requiring hospitalization; HOSP, Hospitalized patients not requiring ventilation; HOSP+O_2_, Hospitalized patients requiring ventilation; ICU, Intensive care unit patients; MOD, moderate patients group; SEV, severe patients group.

A total of 215 patients were included in the Cambridge COVID-19 cohort (CAM), where the moderate group consisted of 56 patients (26.0%) with only mild disease (not requiring hospitalization) and 47 individuals (21.9%) who were hospitalized but did not require additional O_2_ or ventilation. The CAM severe group consisted of 40 hospitalized patients requiring O_2_ (18.6%) and 72 intensive care unit (ICU) patients (33.5%). The distribution of different severity groups of the two cohorts is visualized in **Figure 2**.

Gender distribution was similar in both COVID-19 cohorts (Fisher’s exact test: p>0.9999), while the mean age was higher in the BUD cohort (Mann-Whitney test: p<0.0001). The healthy control cohort consisted of 47 individuals from the CAM cohort and 339 individuals additionally enrolled for genetic analysis in Hungary. Both IL-6 and CRP levels were increased in COVID-19 patients compared to healthy controls, as already shown before (9, 40).

Long COVID, defined as persisting symptoms 6-12 months past infection based on a questionnaire administered to the patients, was observed in 53.3% of the patients enrolled in the Cambridge cohort (32 out of 60 individuals replying to the questionnaire reported persisting symptoms during follow up). Questionnaires regarding persisting symptoms were not performed in the Hungarian cohort.

While 19 out of 215 COVID-19 patients died (8.8%) in the CAM cohort, the prevalence of a COVID-19 related death was significantly higher in the Hungarian cohort (Fisher’s exact test: p=0.007), with a total of 25 non-survivors out of the 128 enrolled cases (19.5%).

### 3.2 The lectin pathway is activated in acute COVID-19

**Figure 3:**
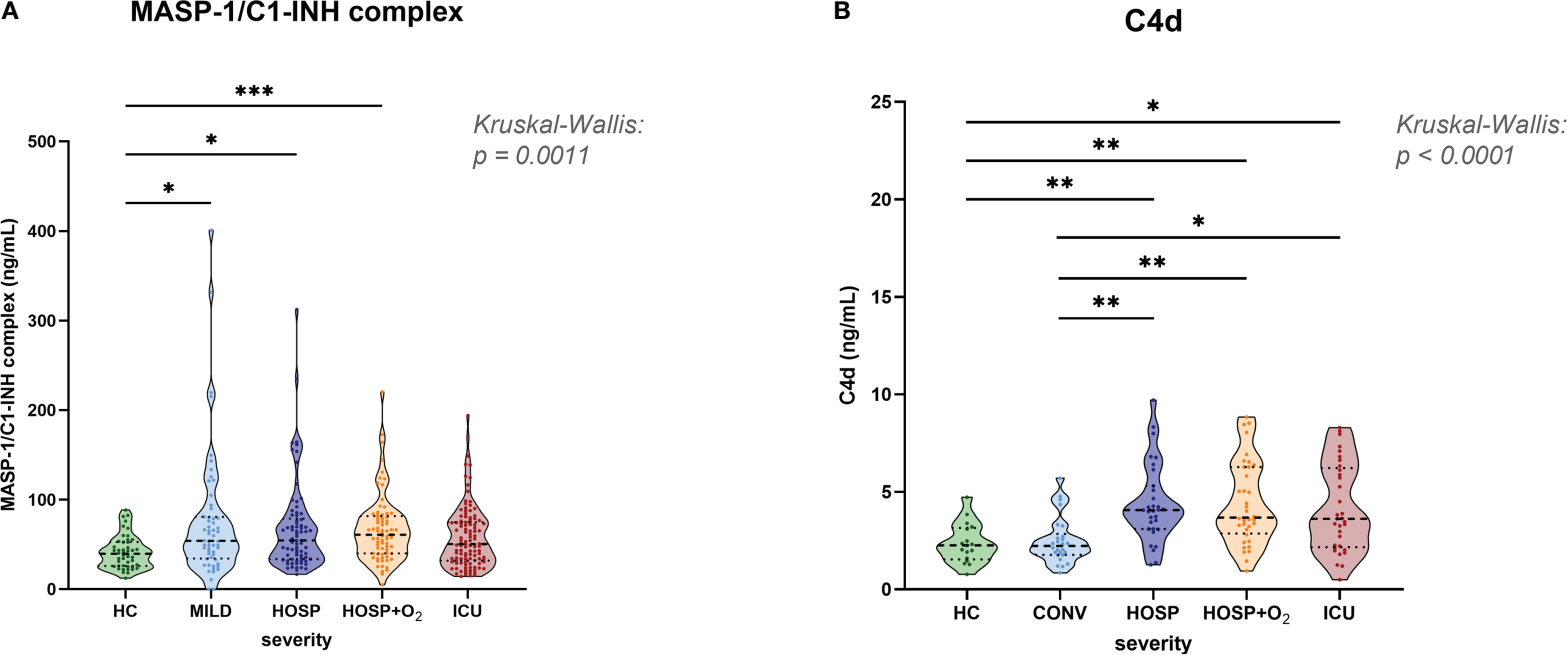
Levels of lectin pathway activation products in healthy controls and COVID-19 patients. **(A)** MASP-1/C1-INH complex and **(B)** C4d levels were measured in EDTA plasma samples and stratified according to severity in healthy controls and the merged COVID-19 cohort. Differences between severity groups were determined using Kruskal-Wallis test with Dunn’s multiple comparison *post-hoc* test. Non-significant differences are not marked, and asterisks indicate significant results (* p<0.05, ** p<0.01, *** p<0.001). HC, Healthy controls; MILD, patients not requiring hospitalization; CONV, patients not requiring hospitalization and sampled during convalescence; HOSP, Hospitalized patients not requiring ventilation; HOSP+O_2_, Hospitalized patients requiring ventilation; ICU, Intensive care unit patients.

Early, specific lectin pathway activation marker MASP-1/C1-INH complex (**Figure 3A**), and C4d (**Figure 3B**), a marker of common CP and LP activation, are elevated in COVID-19 when compared to healthy controls (HC). The median MASP-1/C1-INH complex levels observed in COVID-19 cases were 38% increased (median levels in cases vs. controls: 39.7 vs. 54.5 ng/mL), and C4d levels were 67% elevated when compared to HCs (median levels in cases vs. controls: 2.26 vs. 3.78 ng/mL).

MASP-1/C1-INH complex levels were measured in both cohorts and significantly higher concentrations were observed in hospitalized COVID-19 patients compared to healthy controls (Kruskal-Wallis test: p=0.0008). Levels in the ICU group were slightly lower compared to other hospitalized groups (HOSP and HOSP+O_2_), but differences observed between different hospitalized groups were statistically not significant.

Highly elevated C4d levels could be observed in all hospitalized groups (Kruskal-Wallis test: p<0.0001), while levels of individuals sampled in the convalescent phase (CONV) are in the range of healthy controls (HC). Of note, C4d levels could only be measured in the BUD COVID-19 cohort and a few healthy individuals. Taken together, these results indicate activation of the Lectin pathway in COVID-19.

### 3.3 Correlation patterns between complement markers and laboratory parameters are different in COVID-19 cases and healthy controls

When looking at the correlation between complement markers and laboratory parameters, different correlation patterns are visible in cases vs. controls for both cohorts (see **Supplementary Figure 2**). While several highly significant positive correlations between complement and laboratory parameters can be observed in COVID-19 cases, none of the correlations observed in healthy controls passed correction for multiple testing.

### 3.4 Short or long haplotype combinations of *MBL2* do not associate with COVID-19

Since activation of the lectin pathway is highly dependent on its key pattern recognition molecule MBL, genotyping of the *MBL2* gene was performed for both COVID-19 cohorts as well as for the healthy controls. Analyses were performed according to the short haplotype (only looking at the promoter polymorphism -221 X/Y (rs7096206) and the exonic SNPs A or 0 (missense variants B, C and D)), the respective variant alleles in exon 1 of *MBL2*, as well as according to the long haplotype including all six common polymorphisms. Since haplotype distribution was the same across the two cohorts (data not shown), data from both cohorts were pooled for genetic analysis in order to increase the patient number and hence the statistical power. By pooling, complete genotype data was available from a total of 377 healthy controls and 314 SARS-CoV-2 cases. As summarized in **Tables 2**, **3**, neither the combinations of the short haplotypes nor distribution of the exonic single wildtype and variant alleles of *MBL2* were associated with COVID-19. Separate analysis of the two cohorts are listed in **Supplementary Tables 1–4**.

**Table 2:** Frequencies of short *MBL2* haplotype combinations in healthy controls and cases (merged COVID-19 cohorts (CAM+BUD).

	controls (n=377)		cases (n=314)		Odds ratio (95% Confidence interval)	p-value^a^
	n	%	n	%		
**YA/YA**	102	27.1%	83	26.4%	0.969 (0.695-1.362)	0.864
**YA/XA**	93	24.7%	87	27.7%	1.170 (0.838-1.640)	0.385
**YA/0**	97	25.7%	69	22.0%	0.813 (0.574-1.157)	0.283
**XA/XA**	17	4.5%	13	4.1%	0.915 (0.453-1.853)	0.853
**XA/0**	44	11.7%	41	13.1%	1.137 (0.713-1.801)	0.642
**0/0**	24	6.4%	21	6.7%	1.054 (0.578-1.889)	0.878

a p-values are presented as non-corrected for multiple testing. Threshold for significance taking into account multiple testing is p=0.0083 (Benjamini-Hochberg correction).

**Table 3:** Distribution of exonic wildtype (A) and variant alleles (B, C, and D) of *MBL2* SNPs in healthy controls and cases (merged COVID-19 cohorts (CAM and BUD)).

	controls (n=377)		cases (n=315)		Odds ratio (95% Confidence interval)	p-value^a^
	n	%	n	%		
Wildtype						
**A/A**	**212**	**56.2%**	**183**	**58.1%**	**1.032 (0.761-1.386)**	**0.878**
Heterozygous						
**A/B**	86	22.8%	73	23.2%	1.021 (0.720-1.464)	0.928
**A/C**	7	1.9%	5	1.6%	0.853 (0.304-2.442)	1.000
**A/D**	48	12.7%	33	10.5%	0.802 (0.504-1.296)	0.406
**Total A/0**	**141**	**37.4%**	**111**	**35.2%**	**0.911 (0.671-1.247)**	**0.579**
Homozygous variant						
**B/B**	9	2.4%	8	2.5%	1.066 (0.416-2.610)	1.000
**B/C**	2	0.5%	1	0.3%	0.597 (0.041-5.160)	1.000
**B/D**	9	2.4%	8	2.5%	1.066 (0.416-2.610)	1.000
**C/C**	1	0.3%	1	0.3%	1.197 (0.063-22.810)	1.000
**C/D**	1	0.3%	0	0.0%	0.000 (0.000-10.770)	1.000
**D/D**	2	0.5%	3	1.0%	1.803 (0.366-10.210)	0.664
**Total 0/0**	**24**	**6.4%**	**21**	**6.7%**	**1.051 (0.576-1.883)**	**0.878**

a p-values are presented as non-corrected for multiple testing. Threshold for significance taking into account multiple testing is p=0.0042 (Benjamini-Hochberg correction). Bold values indicate results for the total groups (Wildtype - Heterozygous - Homozygous variant).

**Figure 4:**
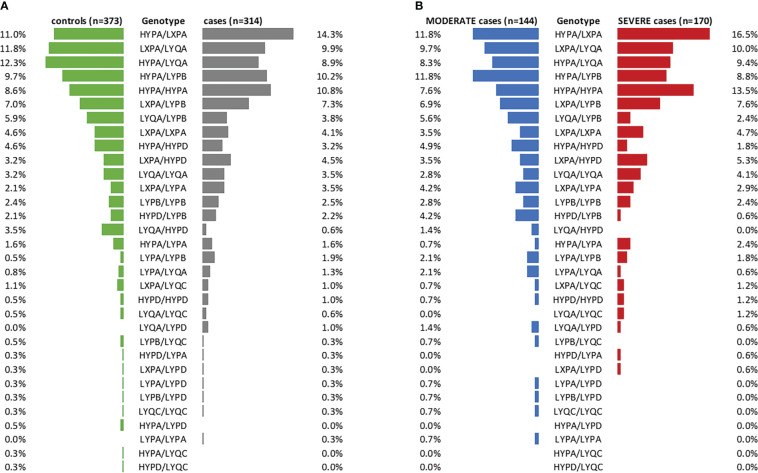
Frequencies of long *MBL2* haplotype combinations in cases and controls. **(A)** Stratification of the merged COVID-19 cohorts (CAM and BUD) according to cases and controls (controls: green, cases: grey). **(B)** Cases stratified according to disease severity (MODERATE: blue, SEVERE: red). n, number.

In addition, no clinically meaningful differences could be observed when looking at the frequencies of long haplotype combinations between cases and controls (visualized in **Figure 4A**, statistical data listed in **Supplementary Table 5**). Only the haplotype combination LYQA/HYPD was more abundant in controls vs. cases (OR=0.178 (95% CI 0.040-0.678), p=0.0155), while a 3.614-fold risk of developing COVID-19 was observed in individuals carrying the LYPA/LYPB haplotype combination (95% CI 0.869-17.700). However, none of the results passed correction for multiple testing (see **Supplementary Table 5**).

Furthermore, distribution of long haplotype combinations also did not differ significantly in moderate COVID-19 cases (n=144, no ventilation required) compared to severe cases (n=170, ventilation and/or ICU treatment required), as visualized in **Figure 4B** (statistical data listed in **Supplementary Table 6**).

Also, when looking at the allelic frequencies, a significant difference between cases and controls was only observed for P/Q (OR=0.758 (95% CI 0.581-0.990), p=0.043), with slightly increased abundance of variant allele Q in controls (22.3%) vs. cases (17.8%), again not passing multiple-testing correction (see **Supplementary Table 7**).

### 3.5 Increased expression of MBL protein and LP activity are genetically determined, but do not strongly associate with COVID-19 severity

**Figure 5:**
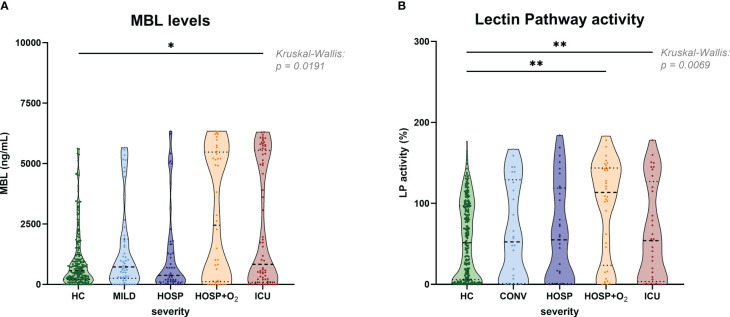
MBL levels (CAM) and Lectin pathway activity (BUD) in healthy controls and COVID-19 patients, stratified according to disease severity. **(A)** MBL levels measured in the Cambridge COVID-19 cohort and healthy individuals and **(B)** Lectin pathway activity measured in the Hungarian COVID-19 cohort and healthy controls stratified according to disease severity. Differences between severity groups were determined using Kruskal-Wallis test with Dunn’s multiple comparison *post-hoc* test. Non-significant differences are not marked, and asterisks indicate significant results (* p<0.05, ** p<0.01). HC, Healthy controls; MILD, patients not requiring hospitalization; CONV, patients not requiring hospitalization and sampled during convalescence; HOSP, Hospitalized patients not requiring ventilation; HOSP+O_2_, Hospitalized patients requiring ventilation; ICU, Intensive care unit patients; LP, lectin pathway.

**Figure 5** shows the measurement of antigenic MBL levels for the CAM cohort (A) and functional lectin pathway activity measured in the BUD cohort (B; adapted from results published before (9)), both stratified according to disease severity. Slightly increased MBL concentrations could be observed in the ICU group compared to healthy controls (HC), while no significant differences were noticed when comparing different COVID-19 severity groups with each other. A similar pattern was observed when looking at the lectin pathway activity in the BUD cohort. Again, significant differences in lectin pathway activity were seen when comparing severe COVID-19 cases (HOSP+O_2_ and ICU group) with healthy controls (HC), but differences in LP activity between individual severity groups were not significant.

**Figure 6:**
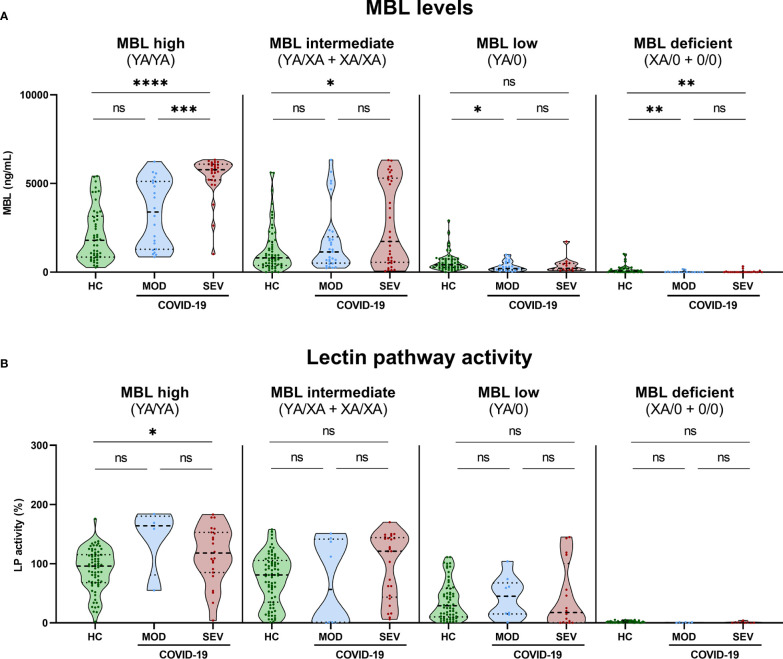
MBL levels (CAM) and Lectin pathway activity (BUD) in healthy controls and COVID-19 patients, stratified according to *MBL2* genotype groups and disease severity. Short *MBL2* haplotype combinations were merged into four different groups: 1) MBL high (YA/YA), 2) MBL intermediate (YA/XA+XA/XA), 3) MBL low (YA/0 (including YA/YB, YA/YC and YA/YD)), and 4) MBL deficient (XA/0+0/0 (including 0/0 for allele A, XA/YB, XA/YC and XA/YD)). After genetic grouping, groups were furthermore stratified according to disease severity, and MBL **(A)** as well as Lectin pathway activity levels **(B)** were compared within groups using ANOVA with Dunn’s multiple comparison *post-hoc* test. Asterisks indicate significant results between severity groups (* p<0.05, ** p<0.01, *** p<0.001, **** p<0.0001). HC, Healthy controls; HOSP, Hospitalized patients not requiring ventilation; HOSP+O_2_, Hospitalized patients requiring ventilation; ICU, Intensive care unit patients; MOD, moderate patients group; SEV, severe patients group; LP, lectin pathway; ns, non-significant.

Grouping of MBL concentrations (**Supplementary Figure 3A**) and Lectin pathway activity (**Supplementary Figure 3B**) of all healthy controls according to the *MBL2* genotype of the individuals did show significant differences between the genotype groups. In addition, both MBL levels and MBL-Lectin pathway activity data was available from 151 healthy controls, and parameters strongly correlated with each other (r=0.7388, p<0.0001; **Supplementary Figure 3C**). When stratified additionally according to disease severity, MBL levels (**Figure 6A**) or LP activity (**Figure 6B**) did differ within the groups too, while patterns are different for MBL high and intermediate (YA/YA and YA/XA+XA/XA) and MBL low or deficient (YA/0 and XA/0+0/0) groups. MBL levels as well as LP activity are higher in COVID-19 cases compared to healthy controls in individuals with YA/YA and YA/XA+XA/XA genotypes. By contrast, slightly higher MBL levels were measured in healthy controls compared to COVID-19 cases in genotypes with variants for the A allele (YA/0 and XA/0+0/0). Individual values of plasma MBL levels as well as lectin pathway activity for each genotype are presented in **Supplementary Figure 4** (A: MBL, B: LP activity).

Those findings indicate that increased MBL protein levels and LP activity are genetically determined by *MBL2* genotype but do not strongly associate with the severity of the disease. However, there seems to be an effect of COVID-19 on the MBL concentration in MBL high and intermediate groups (YA/YA and YA/XA+XA/XA).

### 3.6 Increased MBL protein levels and LP activity are only moderately reflected by increased MASP-1/C1-INH complex and C4d activation product levels

**Figure 7:**
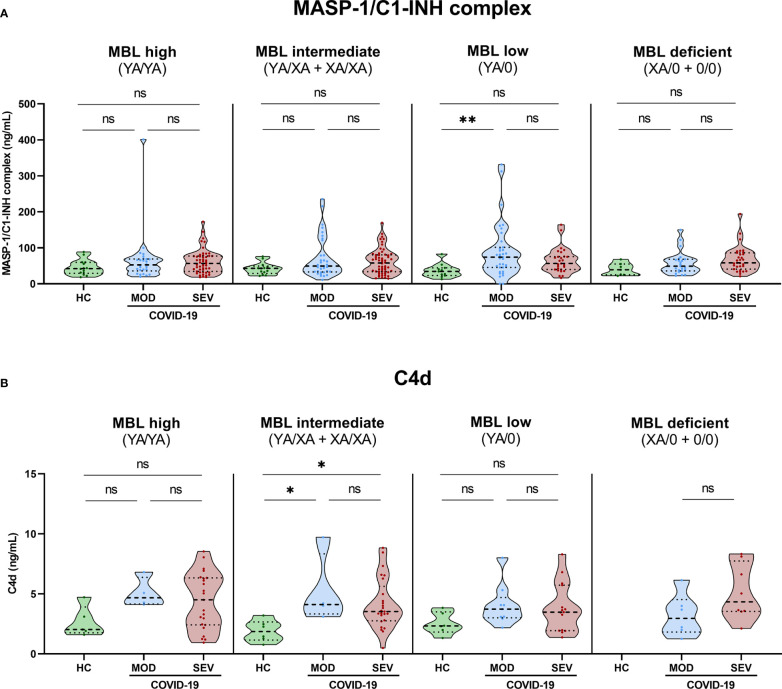
MASP-1/C1-INH complex and C4d levels in healthy controls and COVID-19 patients, stratified according to *MBL2* genotype groups and disease severity. Short *MBL2* haplotype combinations were merged into four different groups: 1) MBL high (YA/YA), 2) MBL intermediate (YA/XA+XA/XA), 3) MBL low (YA/0 (including YA/YB, YA/YC and YA/YD)), and 4) MBL deficient (XA/0+0/0 (including 0/0 for allele A, XA/YB, XA/YC and XA/YD)). After genetic grouping, groups were furthermore stratified according to disease severity, and MASP-1/C1-INH complex **(A)** as well as C4d levels **(B)** were compared within groups using ANOVA with Dunn’s multiple comparison *post-hoc* test. Asterisks indicate significant results between severity groups (* p<0.05, ** p<0.01). HC, Healthy controls; HOSP, Hospitalized patients not requiring ventilation; HOSP+O_2_, Hospitalized patients requiring ventilation; ICU, Intensive care unit patients; MOD, moderate patients group; SEV, severe patients group; ns, non-significant.

Grouping according to *MBL2* genotype and disease severity was also performed for MASP-1/C1-INH complex levels as a marker for ongoing lectin pathway activation (**Figure 7A**) and for C4d concentrations (**Figure 7B**). In both cases, significant differences could only be observed between severity groups and not between different *MBL2* genotype groups, indicating that the increased MBL protein levels and Lectin pathway activity seen in **Figures 5**, **6** are only moderately reflected by increased MASP-1/C1-INH complex and C4d levels, if analysed in homogenous genotype groups.

Individual values of C4d and MASP-1/C1-INH complex levels for each genotype are presented in **Supplementary Figure 5** (A: MASP-1/C1-INH complex, B: C4d).

### 3.7 MBL binds SARS-CoV-2 spike protein in a concentration-dependent manner

**Figure 8:**
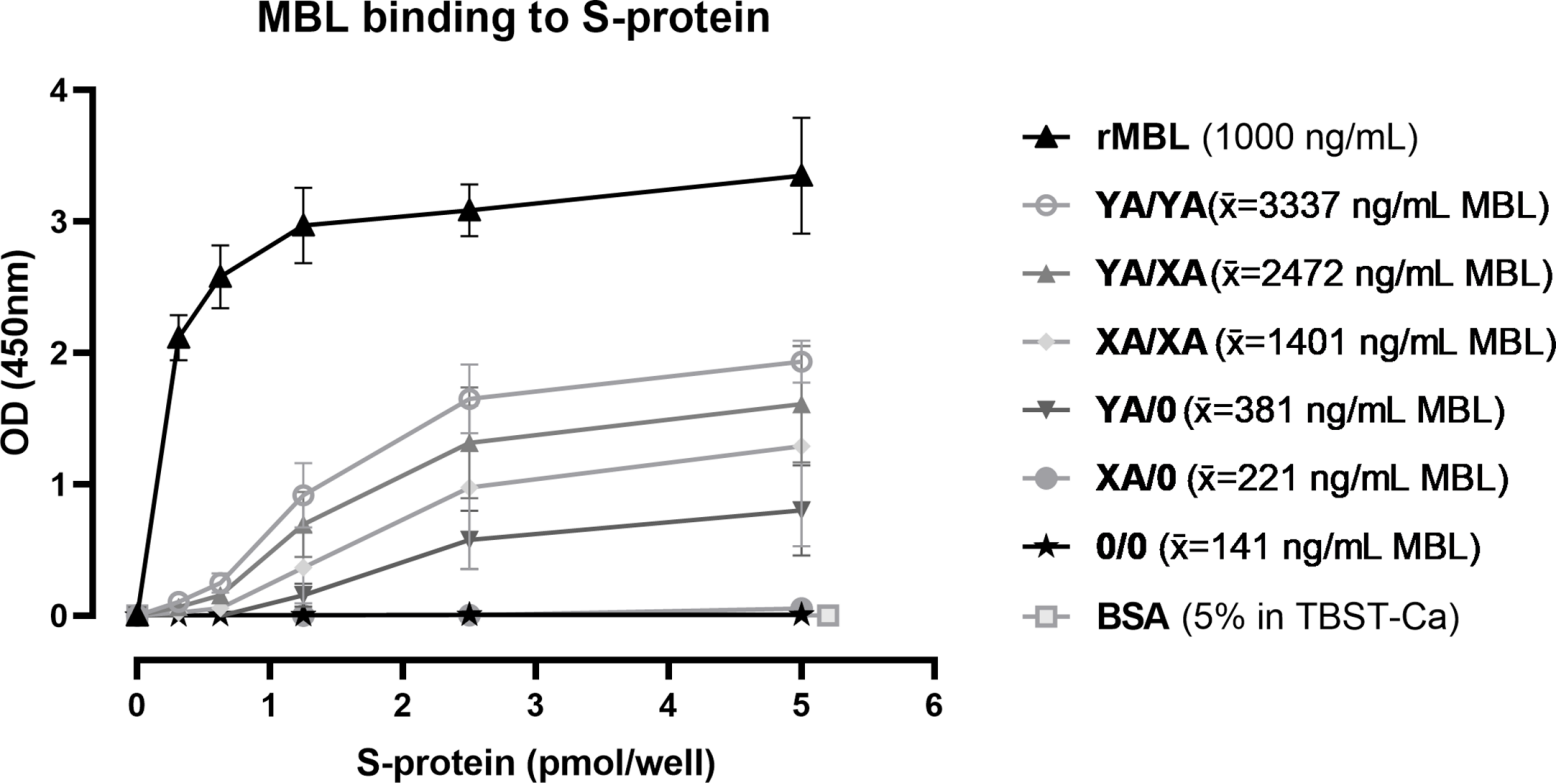
Binding of MBL to SARS-CoV-2 spike protein. Recombinant SARS-CoV-2 spike protein was immobilised on a 96-well plate in different concentrations (0-5 pmol/well). Recombinant MBL (rMBL; 1000 ng/mL), BssSA (5% in TBST-Ca^2+^) or serum from individuals with known *MBL2* genotypes (10% in TBST-Ca^2+^) were incubated on the plates with captured spike protein and bound MBL was detected. Genotypes YA/YA (n=5), YA/XA (n=3), XA/XA (n=3), YA/0 (n=4), XA/0 (n=2) and 0/0 (n=3) were tested, while average MBL levels of the individuals in each group are included in the legend (x̄). Results are presented in mean ± SD. The same results were observed in two independent experiments. OD, optical density; S-protein, SARS-CoV-2 spike protein; rMBL, recombinant MBL; x̄, average concentration; BSA, bovine serum albumin; TBST-Ca, Tris-HCl buffer containing calcium.

SARS-CoV-2 spike protein binding of MBL in serum from individual healthy controls with different genotypes was investigated by ELISA. As shown in **Figure 8**, recombinant MBL (rMBL), as well as MBL present in human serum, was able to bind to the spike protein in a dose-dependent manner, while resulting OD values differ depending on the MBL genotype and therefore MBL antigenic concentrations. Highest signals were obtained when using recombinant MBL, which served as a positive control, while BSA in TBST-Ca^2+^ was used as a negative control and did not show any positive signals. Regarding the human serum samples, highest OD values and hence binding to the spike protein were measured in MBL high/wildtype individuals (YA/YA), followed by YA/XA, XA/XA, and YA/0. Only very low signals were measured in samples from XA/0 individuals, while OD values from sera with 0/0 genotype were on the same level as the negative control (BSA in TBST-Ca^2+^). The trend in OD values follows the average MBL protein levels (x̄) for each genotype group, indicating that binding to the spike protein is only dependent on the MBL levels present in the sera. There is no notable difference between OD values obtained from MBL binding to mannan and binding to the spike protein between different genotypes (data not shown), indicating that the binding behaviour of MBL does not differ from binding to other substrates, when recognizing the SARS-CoV-2 spike protein.

In summary, MBL binds to the SARS-CoV-2 spike protein and the binding is dependent on the MBL protein levels in the serum.

### 3.8 Association between complement markers, mortality, and long-term outcome in COVID-19

**Figure 9:**
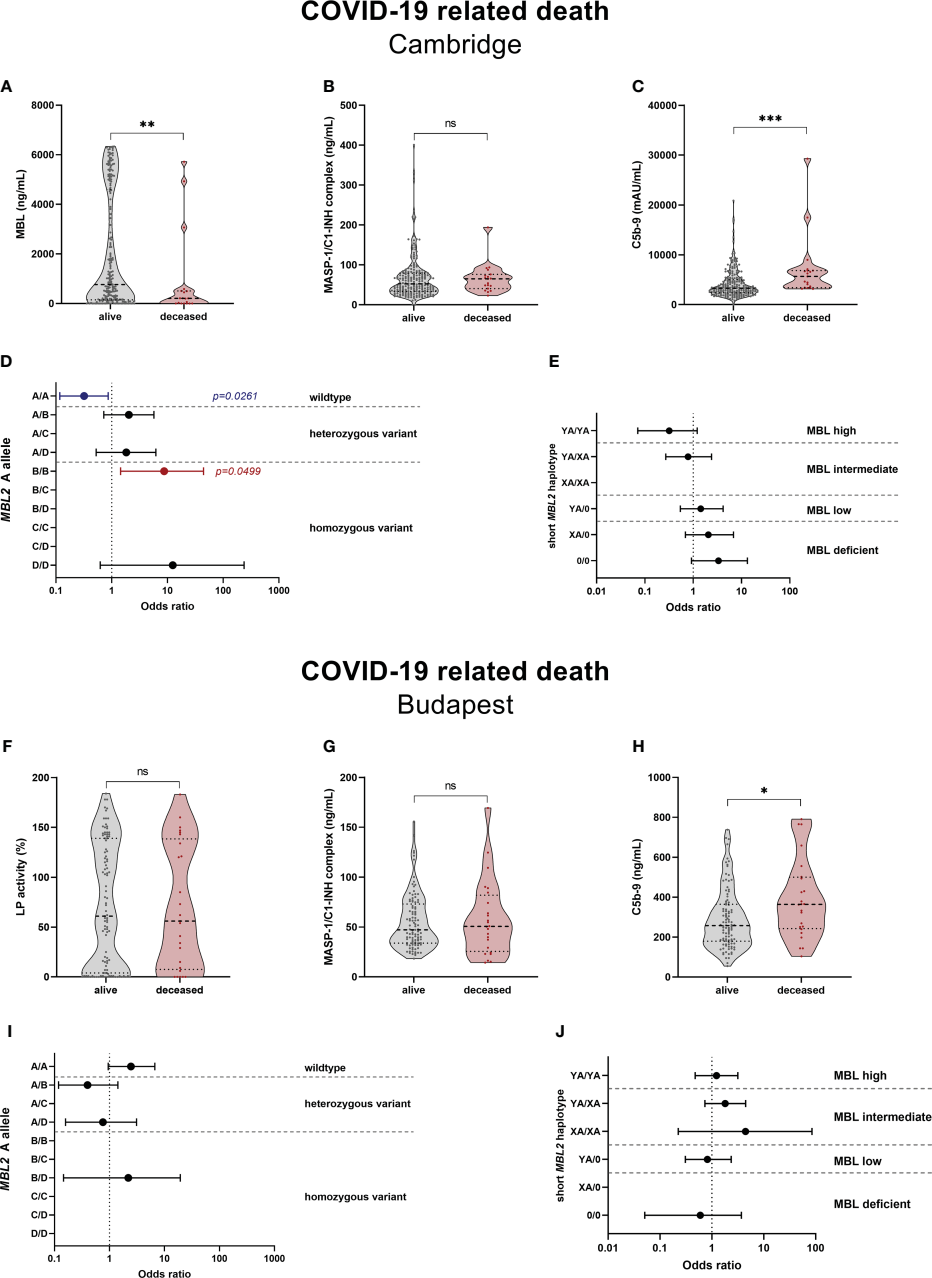
Relationship between complement markers, MBL genotype and mortality in COVID-19. Levels of complement markers MBL **(A)**, MASP-1/C1-INH complex **(B)** and TCC/C5b-9 **(C)** in the Cambridge cohort and Lectin pathway activity **(F)**, MASP-1/C1-INH complex **(G)** and TCC/C5b-9 **(H)** of the Budapest cohort were stratified according to survival (alive vs. deceased). Differences between the two groups were analysed using the Mann-Whitney test, while asterisks indicate significant differences (* p<0.05, ** p<0.01, *** p<0.001). Non-significant differences are indicated (ns). Forest plot displaying Odds ratios (ORs) with 95% confidence intervals (CIs) for COVID-19 related death in individuals, stratified according to the *MBL2* A allele carrier state **(D)** Cambridge, **(E)** Budapest) as well as according to the *MBL2* genotype groups **(I)** Cambridge, **(J)** Budapest). Odds ratios of 0 or infinite are not indicated on the forest plots. p-values are presented as non-corrected for multiple testing. Threshold for significance taking into account multiple testing are p=0.0050 for the *MBL2* A allele, and p=0.0083 for the short *MBL2* haplotype (Benjamini-Hochberg correction). MASP-1/C1-INH, MASP-1/C1-INH complex; LP, lectin pathway activity.

LP-associated complement markers were further analysed for their potential to predict survival and long-term outcome in COVID-19. Both MBL and C5b-9 levels were significantly different between deceased patients and survivors in the Cambridge cohort, with higher MBL (p=0.0062) and lower C5b-9 levels (p=0.0004) in the surviving group, while MASP-1/C1-INH complex levels did not vary between the two groups (p=0.3965) (**Figures 9A–C**). The increased MBL levels in survivors are also reflected by the *MBL2* A allele (**Figure 9D**), where individuals with the MBL high-expressing wildtype allele (A/A) were 70% less likely to die from COVID-19 (OR 0.319 (95% CI 0.117-0.866), p=0.0261), compared to individuals with all other A allele and variant combinations. In contrast, individuals bearing the MBL deficient homozygous variant B/B were >8-times more likely to die from SARS-CoV-2 infection (OR 8.750 (95% CI 1.442-44.470), p=0.0499), compared to individuals with other combinations than B/B. However, the differences seen in the *MBL2* genetics between survivors and non-survivors did not pass correction for multiple testing. Also when looking at the *MBL2* genotype groups (**Figure 9E**), there is a non-significant trend towards decreased ORs in high and intermediate genotype carriers (YA/YA and YA/XA), and increased ORs in carriers of MBL low or deficient genotypes (YA/0, XA/0, 0/0).

In the Hungarian cohort, lectin pathway activity (p=0.8729) and MASP-1/C1-INH complex levels (p=0.9085) did not differ significantly between survivors and deceased patients, while C5b-9 levels were again increased in individuals suffering from a COVID-19 related death (p=0.0186) (**Figures 9F–H**). When looking at the *MBL2* genotypes according to the carrier state of the A allele (**Figure 9I**) and *MBL2* genotype groups (**Figure 9J**), no significant associations with mortality are observed. Since multivariate analysis could not be performed due to low sample size, the distributions of the A allele and short haplotypes are stratified according to disease severity in **Supplementary Tables 8**, **9**.

Since C5b-9 levels were shown to correlate with disease severity in both cohorts (40, 49), and all non-survivors except from one belonged to the severe COVID-19 group (SEV), the comparison was repeated with further stratifying according to the two severity groups. As seen in **Supplementary Figure 6**, disease severity does indeed confound the analysis, since C5b-9 levels did not differ significantly anymore between severely infected survivors (alive) and non-survivors (deceased).

In addition, association with Long COVID as a second outcome was also analysed in the Cambridge cohort. Neither the complement biomarkers investigated (MBL, MASP-1/C1-INH complex, C5b-9), nor the genetic information (carrier state of the A allele or *MBL2* genotype group) were indicative for persisting symptoms 6-12 months after the SARS-CoV-2 infection (**Supplementary Figure 7** and **Supplementary Tables 10**, **11**.

Analysis for outcome was also performed according to the long *MBL2* haplotype combinations; no significant differences were observed (data not shown).

## 4 Discussion

Although by now complement (over)activation is known to play a role in COVID-19, its precise underlying mechanism is not clear. Especially when it comes to the role of the lectin pathway, results are not convincingly supporting firm conclusions. We therefore performed a detailed study looking at lectin pathway activation as well as the six most common SNPs of the *MBL2* gene in two independent cohorts. Our results show that the lectin pathway is indeed activated after COVID-19 onset, while the LP most likely only plays a minor role in the pathogenesis and outcome of the disease. Besides that, the *MBL2* genotype does not greatly affect susceptibility to SARS-CoV-2 infections or disease outcomes such as COVID-19 related mortality and the development of Long COVID.

After the SARS-CoV-1 outbreak in 2002-2004, numerous evidence suggested a key role of MBL in the first-line response against the virus: MBL was shown to bind SARS-CoV and consequently inhibit SARS-CoV S-protein mediated viral infection (50). Besides that, deposition of complement C4 on SARS-CoV was reported to be enhanced by MBL (50). Additionally, a higher frequency of certain low-producing MBL genotypes was found in SARS patients than in controls (51–53). However, those findings could not be replicated in a further study (54). Accordingly, extensive research has been started on the role of MBL in host defence after the current SARS-CoV-2 pandemic. Just recently, Gao et al. demonstrated the ability of the SARS-CoV-2 N protein to bind to MASP-2, thereby leading to MBL-dependent activation of the lectin pathway not only *in vitro*, but also *in vivo* in COVID-19 patients as well as in a mouse model (23). Besides that, Götz et al. showed correlations between MASP-2 protein levels and complement activation as well as inflammatory markers in COVID-19 (55), also pointing towards LP activation in the disease course. Niederreiter et al. showed marked complement activation *via* the lectin and alternative pathway in the lungs of patients deceased from COVID-19, while in kidneys only LP activation (measured by the deposition of MASP-2) was observed (56). Here, we present results including two cohorts of SARS-CoV-2 infected patients. The first cohort was enrolled at Addenbrooke’s and Royal Papworth’s Hospital in Cambridge, UK. In a prior study, it was shown that severe COVID-19 could be distinguished from mild disease through delayed bystander CD8^+^ T cell activation and early immune pathology (40). Furthermore, the study showed associations of both cytokine levels and complement components with follow-up time and disease severity, with higher levels of C3 activation products (C3a and C3c) and the terminal complement complex (TCC/C5b-9) in more severe cases (40). Increased complement activation was also characteristic for severe SARS-CoV-2 infections in the second cohort included in the analysis, sampled at two tertiary care hospitals in Budapest, Hungary. Severe COVID-19 cases were more likely to die when the infection went along with over-activation of complement and consumption of C3 (9).

In our present analysis, we could show that levels of both the MASP-1/C1-INH complex, a new biomarker for early lectin pathway activation (described in detail in (46)), and the joint LP/CP marker C4d were increased in more severe cases of COVID-19, indicating activation of the lectin pathway during SARS-CoV-2 infection. However, MASP-1/C1-INH complex levels did not differ significantly between the ICU group and healthy controls. We speculate, that it is most likely caused by additional complications in the ICU patients, such as renal failure, severe pneumonia, or embolism, as well as an exhausted immune response with complement consumption (declining C3, C4, AP and CP activity, as also shown in our previous publication (9). Nevertheless, MASP-1/C1-INH complex levels positively correlate with C4d and TCC/C5b-9 concentrations in COVID-19 cases in general, further validating the usage of MASP-1/C1-INH complex as a direct measure of early lectin pathway activation.

During SARS-CoV-2 infection, the lectin pathway can be activated directly through interaction of viral proteins and the LP pattern recognition molecules. In a recent study, Stravalaci et al. could not demonstrate binding of MBL to the N protein, but verified its ability to recognize the S protein and to consequently inhibit SARS-CoV-2 entry into host cells (26), caused by the high glycosylation of the spike protein (57, 58). Binding of MBL to the SARS-CoV-2 spike protein was also confirmed in our study, where we did investigate binding of not only recombinant MBL (rMBL) to the viral protein, but also included native MBL present in human sera from healthy individuals with different *MBL2* genotypes. Similar to recombinant MBL, also MBL from human sera was able to bind to the spike protein, while the binding was only dependent on the MBL concentration in the serum. These findings, combined with the results from the Stravalaci group (26), suggest a protective role of MBL in the early course of COVID-19.

A protective role of MBL during SARS-CoV-2 infections can further be strengthened by investigating the *MBL2* single nucleotide polymorphisms. Medetalibeyoglu et al. reported an association of the MBL deficient B/B genotype with COVID-19 (59). Moreover, they showed that individuals bearing the B allele have a higher risk of developing a more severe clinical phenotype and ICU need, but not of higher mortality (59). Similarly, Speletas et al. showed that carrying the *MBL2* B allele predisposes COVID-19 patients to a worse disease course with an increased risk of hospitalization and development of pneumonia (60). In their studies, no similar effect was found either for the C, D, or X alleles, or any haplotypes. Also Stravalaci and colleagues found an increased risk of disease development in individuals bearing two of the exonic disruptive variants by comparing severe COVID-19 cases and controls (26). Correspondingly, Hultström et al. performed extensive genetic studies in a multicentre cohort of severely infected COVID-19 cases, further including publicly available genetic data from the COVID-19 Human Genetics Initiative (39). In their study, genetic *MBL2* variants were not associated with the need for hospitalization or ICU admission during SARS-CoV-2 infection, but haplotype combinations with intermediate MBL expression (LXA/LXA, HYA/0, LYA/0) were found to be protective when it comes to thromboembolic complications in critically ill COVID-19 patients (39). In this analysis, providing information from two independent cohorts, we could not find any association between genetic variations in *MBL2* and the risk of SARS-CoV-2 infection, when investigating distributions of either short or long *MBL2* haplotype combinations or exonic wildtype (A) and variant alleles between COVID-19 cases and controls. Intriguingly, we observed a rather “protective” role for MBL in the risk of a COVID-19 related death in one of the cohorts (the CAM cohort). Survivors showed higher MBL levels than deceased patients, which was not confounded by disease severity (as seen in **Supplementary Figure 6A**). In addition, also the wildtype *MBL2* A alleles (A/A) were associated with a lower risk of dying from COVID-19, while individuals bearing the B/B combination seem to be at a higher risk to die from COVID-19. However, the findings in the genetic analysis did not pass correction for multiple testing and could not be replicated in our second cohort (BUD), which might be caused by the sample size. Nevertheless, other studies reported an association of the B allele with a higher risk for a more severe disease course following SARS-CoV-2 infection, but not mortality (59, 60). Although these interesting findings in our subgroups lack power, they clearly highlight the necessity for confirmatory/follow up studies including larger cohorts or meta-analysis in the future. Additional discrepancies in the two cohorts might be explained by disease-severity related heterogeneity (more severe cases in the Hungarian cohort, with worse outcome) or by differences in the patient care and sample handling (e.g. different thresholds for hospital admission or variance in the capacity of intensive care units). Furthermore, some studies included additional SNPs spanning the entire *MBL2* region in their analysis (26, 61), while others did not (39), so inconsistencies in the way of analysing the data might also have an impact on the results.

When investigating Long COVID as a secondary outcome, neither the measured biomarkers nor the *MBL2* variants pointed towards a role of the lectin pathway in the development of Long COVID, defined as persisting symptoms 6-12 months after the SARS-CoV-2 infection. On the other hand, high MBL levels, and hence activation of the lectin pathway, were suggested to contribute to thromboembolic complications in severe COVID-19 patients (12). Holter et al. showed similar MBL plasma levels in COVID-19 patients and controls, with only a modest increase at days 3 to 5 after hospital admission (62). In our study, we can verify slightly increased MBL levels in more severe COVID-19 patients when compared to healthy controls. Those results are also visible within different *MBL2* genotype groups, suggesting that COVID-19 patients with MBL high (YA/YA) and MBL intermediate (YA/XA+XA/XA) *MBL2* genotypes can increase MBL levels upon infection, while that is not possible for MBL low and deficient genotypes (YA/0 and XA/0+0/0). Although this effect is less pronounced when looking at the lectin pathway activity, there is still a trend in the same direction. MBL is suggested to act as an acute phase protein during infections (63), although debated extensively. While MBL levels did not change as a result of an acute phase reaction caused by surgery (64), others found increasing MBL concentrations during acute phase responses (65, 66). Similar to results published in children with malignancy (67) and sepsis patients (66), we were able to observe increased MBL levels in COVID-19 individuals with wildtype *MBL2* genes in our study, while this was not the case for individuals from the MBL low+deficient groups (**Figure 6A**). A potential mechanism for the increased MBL levels in more severe cases, even within genotype groups, could potentially be explained by the promoter effect. SARS-CoV-2 infection may increase MBL expression in more severe SARS-CoV-2 infections by increased IL-6 secretion. *In vitro*, it was shown that MBL expression can be regulated by IL-6 with a 2-3 fold increase, while there was no effect on MBL expression by other cytokines such as TNFa and IFNy (68). As published earlier, IL-6 levels were significantly increased in more severe COVID-19 patients in both cohorts analysed (9, 40), which subsequently could increase expression of MBL.

Of note, the lectin pathway activity (LP%) was determined utilizing the WIESLAB^®^ Complement System MBL Pathway kit. While the assay can only measure MBL-induced lectin pathway activity, there are several other pattern recognition molecules able to give rise to lectin pathway activation (15–17). Although Stravalaci et al. were not able to show binding of the SARS-CoV-2 spike protein to either ficolins or other collectins than MBL (26), Ali et al. demonstrated binding of MBL, ficolin-2 and collectin-11 to the SARS-CoV-2 S and N proteins (25). Even if we did not measure those alternative PRMs in the scope of our study, our results suggest that they might still be able to increase lectin pathway activation, resulting in increased levels of MASP-1/C1-INH complex and C4d in COVID-19 patients.

Our study does have several limitations. First of all, not all measurements could be performed in all individuals due to restriction in sample availability, which is especially true for biomarker determinations (C4d, MBL, MBL-lectin pathway activity). However, similar results could be observed when either looking at MBL levels or MBL-lectin pathway activity in most cases. Both, MBL antigenic levels and LP% from the same individuals was available from a total of 151 healthy controls of the BUD cohort, and values strongly correlated with each other. Besides that, different assays/methods were used for some determinations in the two cohorts (TCC/C5b-9), not allowing us to merge the results for all analysis performed. Some individuals also had to be excluded since either genetic or biomarker data (MBL/LP activity) were missing, or because sampling was performed in convalescence and measured biomarker values could therefore not be included in the analysis. In addition, the study design does not allow us to draw firm conclusions regarding disease susceptibility, since different exposures likely account for susceptibility to COVID-19 much more than genetic predisposition and the included healthy controls were not matched with COVID-19 patients in terms of exposure to SARS-CoV-2.

However, results obtained are similar in both cohorts and for large parts of the analysis the two independent cohorts could be merged, making it an extensive study of lectin pathway activation in COVID-19 with higher number of individuals compared to other such studies. In comparison to others, we did not only focus on either genetic variants or protein levels, but instead investigated LP activation in SARS-CoV-2 with an integrated approach, including the six common *MBL2* polymorphisms, relevant protein levels, MBL-lectin pathway activity and several respective activation products. While all our conclusions are based on biomarker and activity measurements from samples taken on admission, there is the chance that we might miss out on interesting associations occurring later on during the disease course. Complications, such as thromboembolic events, might also be related to lectin pathway activation, and hence follow-up samples might shed further light on the role of lectin LP activation in COVID-19. Another interesting point for the future might be to investigate data from different waves of the pandemic, since all results shown here are referring to samples collected within the first wave of the pandemic.

## 5 Conclusion

In conclusion, we showed that the lectin pathway is activated during SARS-CoV-2 infection, with higher activation in more severe cases of COVID-19. MBL is binding to the SARS-CoV-2 spike protein in an MBL concentration dependent manner, while *MBL2* genotypes do not further influence the binding. However, activation of MBL-LP only plays a minor role in COVID-19 pathogenesis, since no clinically meaningful, consistent associations with disease outcomes were noted. Besides that, no strong association between genetic variations in *MBL2* and the risk of SARS-CoV-2 infection was observed.

## Data availability statement

The datasets presented in this study can be found in online repositories. The names of the repository/repositories and accession number(s) can be found below: ERZ16293917 and ERZ16293918 (Project: PRJEB60441), accessible under following link: https://www.ebi.ac.uk/eva/?eva-study=PRJEB60441.

## Ethics statement

The studies involving human participants were reviewed and approved by Hungarian Ethical Review Agency (ETT-TUKEB), the Government Office of the Capital City Budapest (31110-7/2014/EKU (481/2014)), the East of England – Cambridge Central Research Ethics Committee (“NIHR BioResource” REC ref 17/EE/0025, and “Genetic variation AND Altered Leucocyte Function in health and disease – GANDALF” REC ref 08/H0308/176). The patients/participants provided their written informed consent to participate in this study.

## Cambridge Institute of Therapeutic Immunology and Infectious Disease-National Institute of Health Research (CITIID-NIHR) COVID BioResource Collaboration

Stephen Baker, John R. Bradley, Patrick F. Chinnery, Daniel J. Cooper, Gordon Dougan, Ian G. Goodfellow, Ravindra K. Gupta, Nathalie Kingston, Paul J. Lehner, Paul A. Lyons, Nicholas J. Matheson, Caroline Saunders, Kenneth G. C. Smith, Charlotte Summers, James Thaventhiran, M. Estee Torok, Mark R. Toshner, Michael P. Weekes, Gisele Alvio, Sharon Baker, Areti Bermperi, Karen Brookes, Ashlea Bucke, Jo Calder, Laura Canna, Cherry Crucusio, Isabel Cruz, Rnalie de Jesus, Katie Dempsey, Giovanni Di Stephano, Jason Domingo, Anne Elmer, Julie Harris, Sarah Hewitt, Heather Jones, Sherly Jose, Jane Kennet, Yvonne King, Jenny Kourampa, Emily Li, Caroline McMahon, Anne Meadows, Vivien Mendoza, Criona O’Brien, Charmain Ocaya, Ciro Pascuale, Marlyn Perales, Jane Price, Rebecca Rastall, Carla Ribeiro, Jane Rowlands, Valentina Ruffolo, Hugo Tordesillas, Phoebe Vargas, Bensi Vergese, Laura Watson, Jieniean Worsley, Julie-Ann Zerrudo, Laura Bergamaschi, Ariana Betancourt, Georgie Bower, Ben Bullman, Chiara Cossetti, Aloka De Sa, Benjamin J. Dunore, Maddie Epping, Stuart Fawke, Stefan Gräf, Richard Grenfell, Andrew Hinch, Josh Hodgson, Christopher Huang, Oisin Huhn, Kelvin Hunter, Isobel Jarvis, Emma Jones, Maša Josipović, Ekaterina Legchenko, Daniel Lewis, Joe Marsden, Jennifer Martin, Federica Mescia, Francesca Nice, Ciara O’Donnell, Ommar Omarjee, Marianne Perera, Linda Pointon, Nicole Pond, Nathan Richoz, Nika Romashova, Natalia Savoinykh, Rahul Sharma, Joy Shih, Mateusz Strezlecki, Rachel Sutcliffe, Tobias Tilly, Zhen Tong, Carmen Treacy, Lori Turner, Jennifer Wood, Marta Wylot, John Allison, Heather Biggs, Helen Butcher, Daniela Caputo, Debbie Clapham-Riley, Eleanor Dewhurst, Christian Fernandez, Anita Furlong, Barbara Graves, Jennifer Gray, Tasmin Ivers, Emma Le Gresley, Rachel Linger, Mary Kasanicki, Sarah Meloy, Francesca Muldoon, Nigel Ovington, Sofia Papadia, Christopher J. Penkett, Isabel Phelan, Venkatesh Ranganath, Jennifer Sambrook, Katherine Schon, Hannah Stark, Kathleen E. Stirrups, Paul Townsend, Julie von Ziegenweidt, Jennifer Webster, Ali Asaripour, Lucy Mwaura, Caroline Patterson, Gary Polwarth, Katherine Bunclark, Michael Mackay, Alice Michael, Sabrina Rossi, Mayurun Selvan, Sarah Spencer, Cissy Yong, Petra Polgarova

## Author contributions

ZP, PL, ET, and GS designed, conceptualized and supervised the study. ZP, PL, FM, LB, GS, MR, VM, ZI, JG, LG, PR, BS, BL, JS, IB, ZZP, ZF, TM, and IV-N were involved in recruitment and sampling of healthy controls and COVID-19 patients as well as in the collection of clinical data. LH, ÁS, FM, LB, BM, DC, and EK performed experiments, and analysed and interpreted the data. ZP, ET, PL, RW, and LC analysed and interpreted clinical and experimental data. LH and ÁS drafted the manuscript. ZP, ET, PL, GS, RW, BM, VM, DC, EK, PK, FM, LB, and LC critically reviewed the manuscript. All authors contributed to the article and approved the submitted version.
