# Supplementary material for: Complement lectin pathway activation is associated with COVID-19 disease severity, independent of MBL2 genotype subgroups
Source: Front Immunol. 2023 Mar 27;14:1162171. doi: 10.3389/fimmu.2023.1162171 (PMC10084477; doi:10.3389/fimmu.2023.1162171)
Supplement: Supplementary file 1 [file DataSheet_1.pdf]

Supplementary Material

Complement lectin pathway activation is associated with COVID-19 disease severity, independent of *MBL2* genotype subgroups

Lisa Hurler [1], Ágnes Szilágyi [1], Federica Mescia [2,3], Laura Bergamaschi [2,3], Blanka Mező [1,4], György Sinkovits [1], Marienn Réti [5], Veronika Müller [6], Zsolt Iványi [7], János Gál [7], László Gopcsa [5], Péter Reményi [5], Beáta Szathmáry [8], Botond Lakatos [8], János Szlávik [8], Ilona Bobek [9], Zita Z. Prohászka [1], Zsolt Förhécz [1], Dorottya Csuka [1], Erika Kajdácsi [1], László Cervenak [1], Petra Kiszel [4], Tamás Masszi [1], István Vályi-Nagy [5], Reinhard Würzner [10], Cambridge Institute of Therapeutic Immunology and Infectious Disease-National Institute of Health Research (CITIID-NIHR) COVID BioResource Collaboration, Paul A. Lyons [2,3], Erik J. M. Toonen [11], Zoltán Prohászka [1,4,*]

[1] Department of Internal Medicine and Haematology, Semmelweis University, Budapest, Hungary

[2]Cambridge Institute of Therapeutic Immunology and Infectious Disease, Jeffrey Cheah Biomedical Centre, University of Cambridge, Cambridge, UK

[3]Department of Medicine, University of Cambridge, Addenbrooke's Hospital, Cambridge, UK

[4]Research Group for Immunology and Haematology, Semmelweis University - Eötvös Loránd Research Network (Office for Supported Research Groups), Budapest, Hungary

[5] Department of Haematology and Stem Cell Transplantation, Central Hospital of Southern Pest - Institute of Haematology and Infectious Diseases, Budapest, Hungary

[6] Department of Pulmonology, Semmelweis University, Budapest, Hungary

[7] Department of Anaesthesiology and Intensive Therapy, Semmelweis University, Budapest, Hungary

[8] Department of Infectology, Central Hospital of Southern Pest - Institute of Haematology and Infectious Diseases, Budapest, Hungary

[9] Department of Anaesthesiology and Intensive Therapy, Central Hospital of Southern Pest - Institute of Haematology and Infectious Diseases, Budapest, Hungary

[10]Institute of Hygiene and Medical Microbiology, Medical University of Innsbruck, Innsbruck, Austria

[11]Research and Development Department, Hycult Biotech, Uden, The Netherlands

[*] **Correspondence:** Zoltán Prohászka, prohaszka.zoltan@med.semmelweis-univ.hu

SUPPLEMENTARY MATERIAL

1 Supplementary Figures

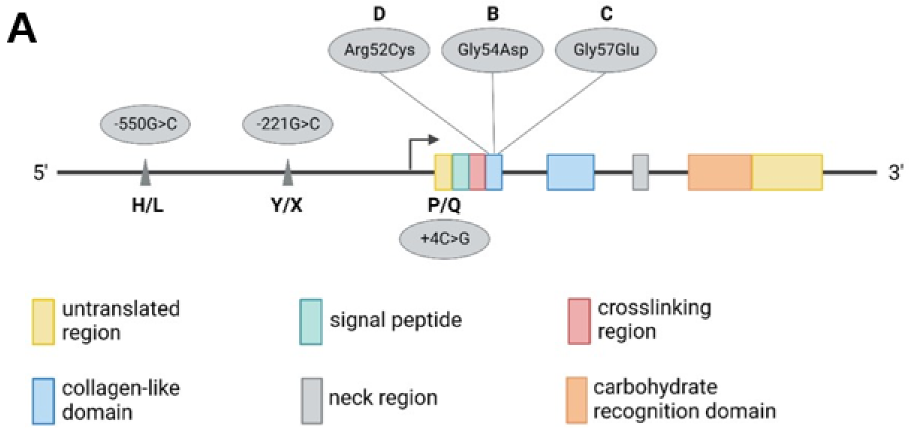

B

polymorphism	gene position	rs ID	Chromosome position	Amino acid substitution
**X → Y**	promoter	rs7096206	chr10:52771925:G:C	
**A → B**	exon 1	rs1800450	chr10:52771475:C:T	Gly54Asp
**A → C**	exon 1	rs1800451	chr10:52771466:C:T	Gly57Glu
**A → D**	exon 1	rs5030737	chr10:52771482:G:A	Arg52Cys
**H → L**	promoter	rs11003125	chr10:52772254:G:C	
**P → Q**	5' UTR	rs7095891	chr10:52771701:G:A	

C

Short genotype	MBL expression
**YA/YA**	MBL high/wildtype
**YA/XA**<br>**XA/XA**	MBL intermediate
**YA/0**	MBL low
**XA/0**<br>**0/0**	MBL deficient

Supplementary Figure 1: Overview of the six common *MBL2* single nucleotide polymorphisms. (A) Overview of the *MBL2* gene structure. The gene consists of 4 exons and locations of the six common SNPs investigated here are indicated in the gene structure in circles. Figure created with BioRender.com. (B) Summary of the six major *MBL2* single nucleotide polymorphisms. (C) Genotype groups of *MBL2* as used in the analysis and associated MBL protein levels.

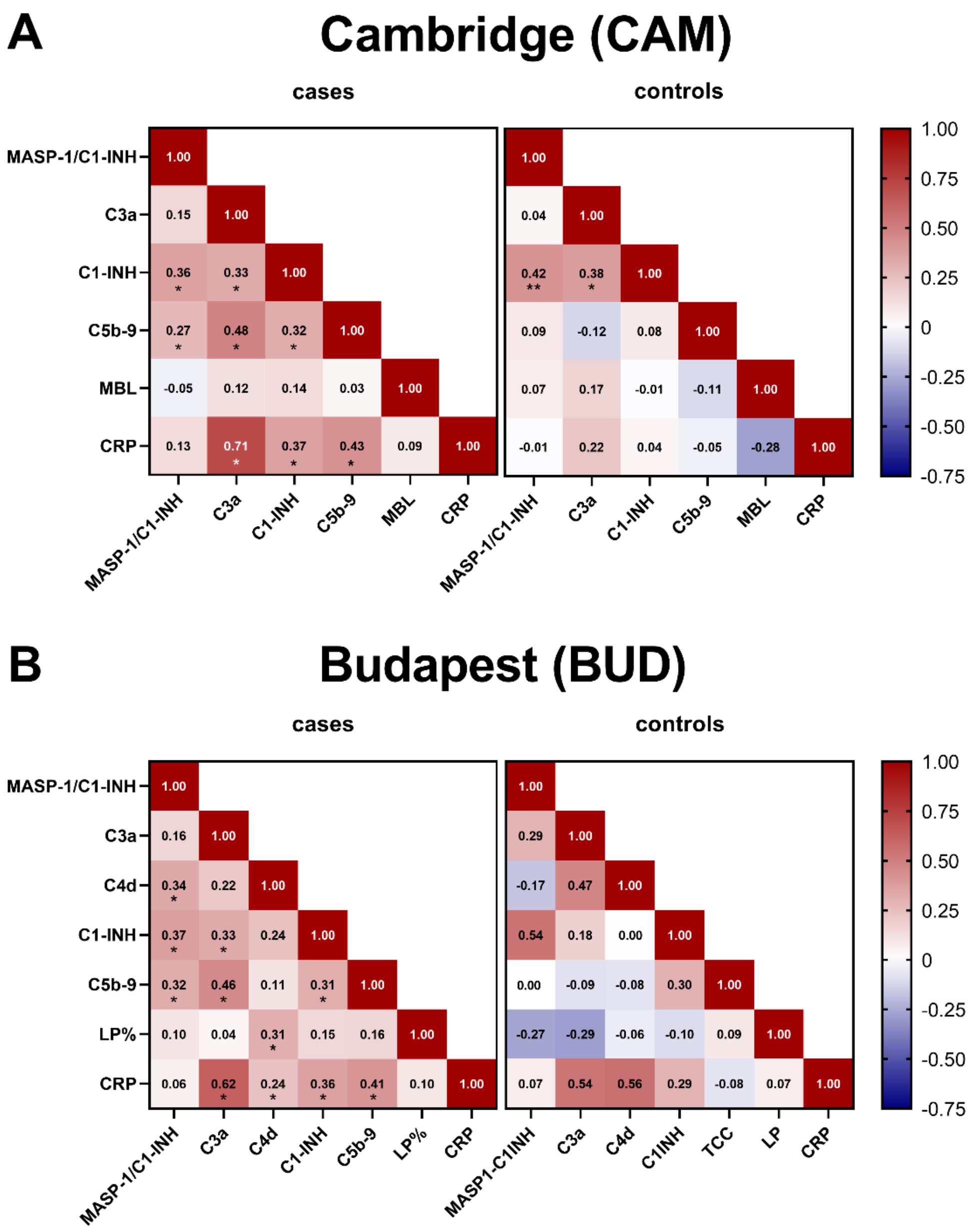

Supplementary Figure 2: Correlation between complement and laboratory parameters in cases and controls. Heatmap of correlation matrix of complement and inflammatory markers in the Cambridge cohort (A, CAM) and the Budapest cohort (B, BUD). Correlations in cases are shown in the left panel each, while correlations in controls are shown on the right. Color-coding indicates the strength of each correlation (Spearman correlation coefficients) with asterisks indicating significance after 5% false discovery rate correction using the Benjamini-Hochberg method.

Abbreviations: C5b-9, terminal complement complex; LP, lectin pathway activity; CRP, C-reactive protein.

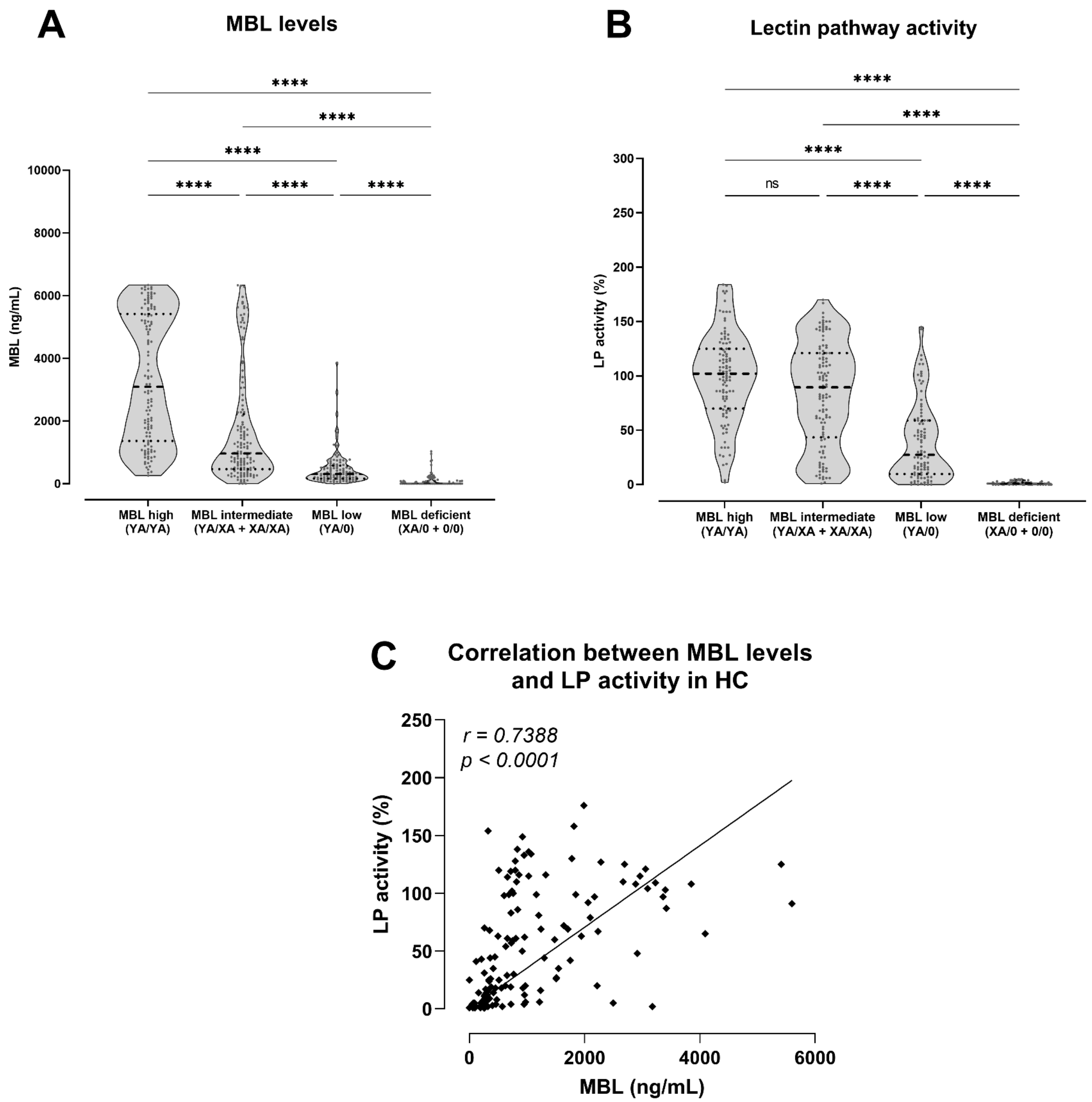

Supplementary Figure 3: MBL levels (A) and Lectin pathway activity (B) stratified according to *MBL2* genotype groups as well as correlation between the two markers (C). MBL levels and Lectin pathway activity were stratified according to short *MBL2* haplotype combinations into four different groups: 1) YA/YA, 2) YA/XA+XA/XA, 3) YA/0 (including YA/YB, YA/YC and YA/YD), and 4) XA/0+0/0 (including 0/0 for allele A, XA/YB, XA/YC and XA/YD). Differences between genetic groups were analysed using Kruskal-Wallis test with Dunn's multiple comparison post-hoc test. Correlation of MBL levels and LP activity were tested using the Spearman r correlation. Asterisks indicate significant results (* p<0.05, ** p<0.01, *** p<0.001, **** p<0.0001).

Abbreviations: LP, lectin pathway; ns, not significant.

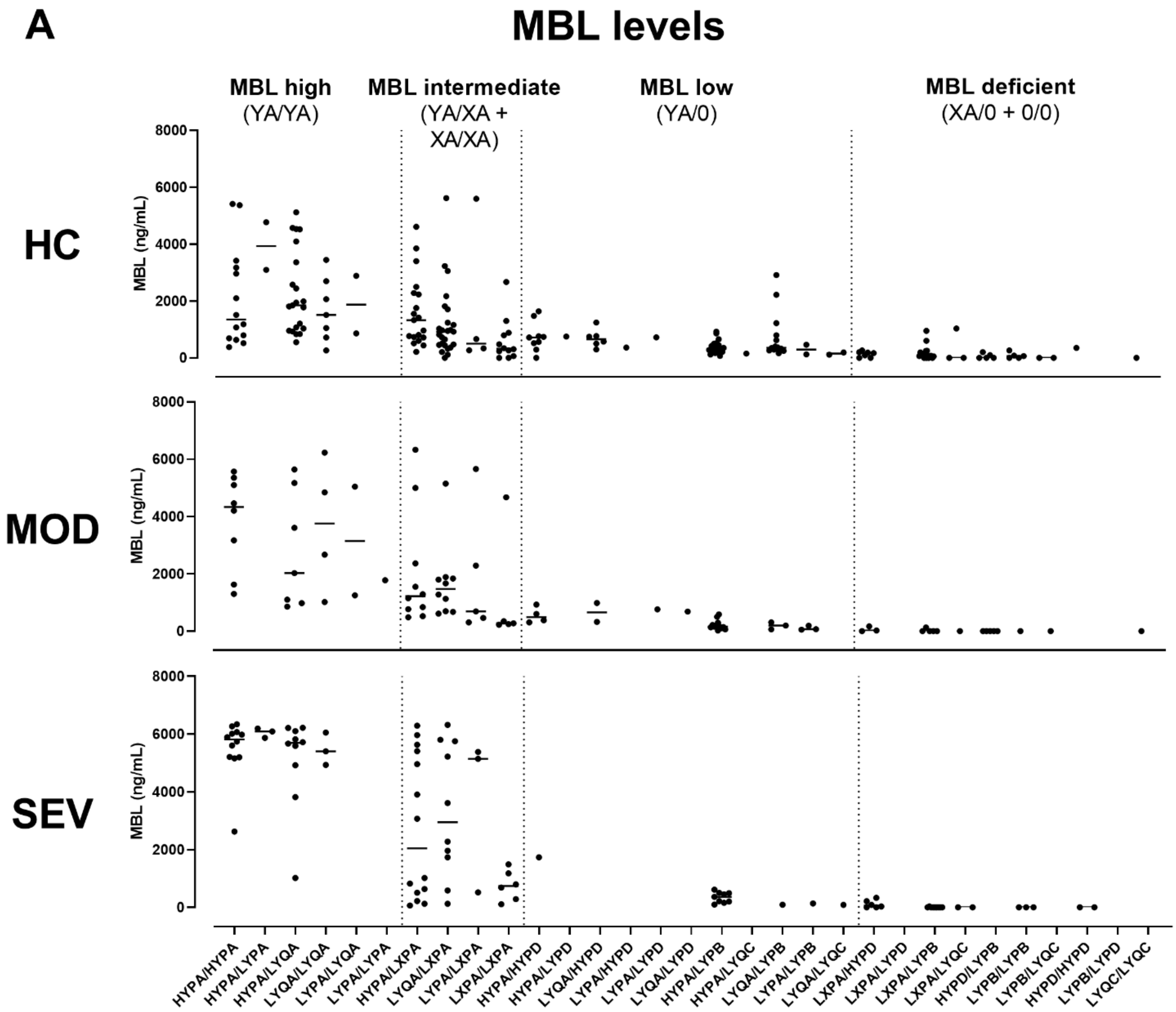

A
MBL levels
MBL high (YA/YA)
MBL intermediate (YA/XA + XA/XA)
MBL low (YA/0)
MBL deficient (XA/0 + 0/0)
HC
MOD
SEV
MBL (ng/mL)
8000
6000
4000
2000
0
HYPA/HYPA
HYPA/LYPA
HYPA/LYQA
LYQA/LYQA
LYPA/LYQA
LYPA/LYPA
HYPA/LXPA
LYQA/LXPA
LYPA/LXPA
LXPA/LXPA
HYPA/HYPD
HYPA/LYPD
LYQA/HYPD
LYPA/HYPD
LYPA/LYPD
LYQA/LYPD
HYPA/LYPB
HYPA/LYQC
LYQA/LYPB
LYPA/LYPB
LYQA/LYQC
LXPA/HYPD
LXPA/LYPD
LXPA/LYPB
LXPA/LYQC
HYPD/LYPB
LYPB/LYPB
LYPB/LYQC
HYPD/HYPD
LYPB/LYPD
LYQC/LYQC

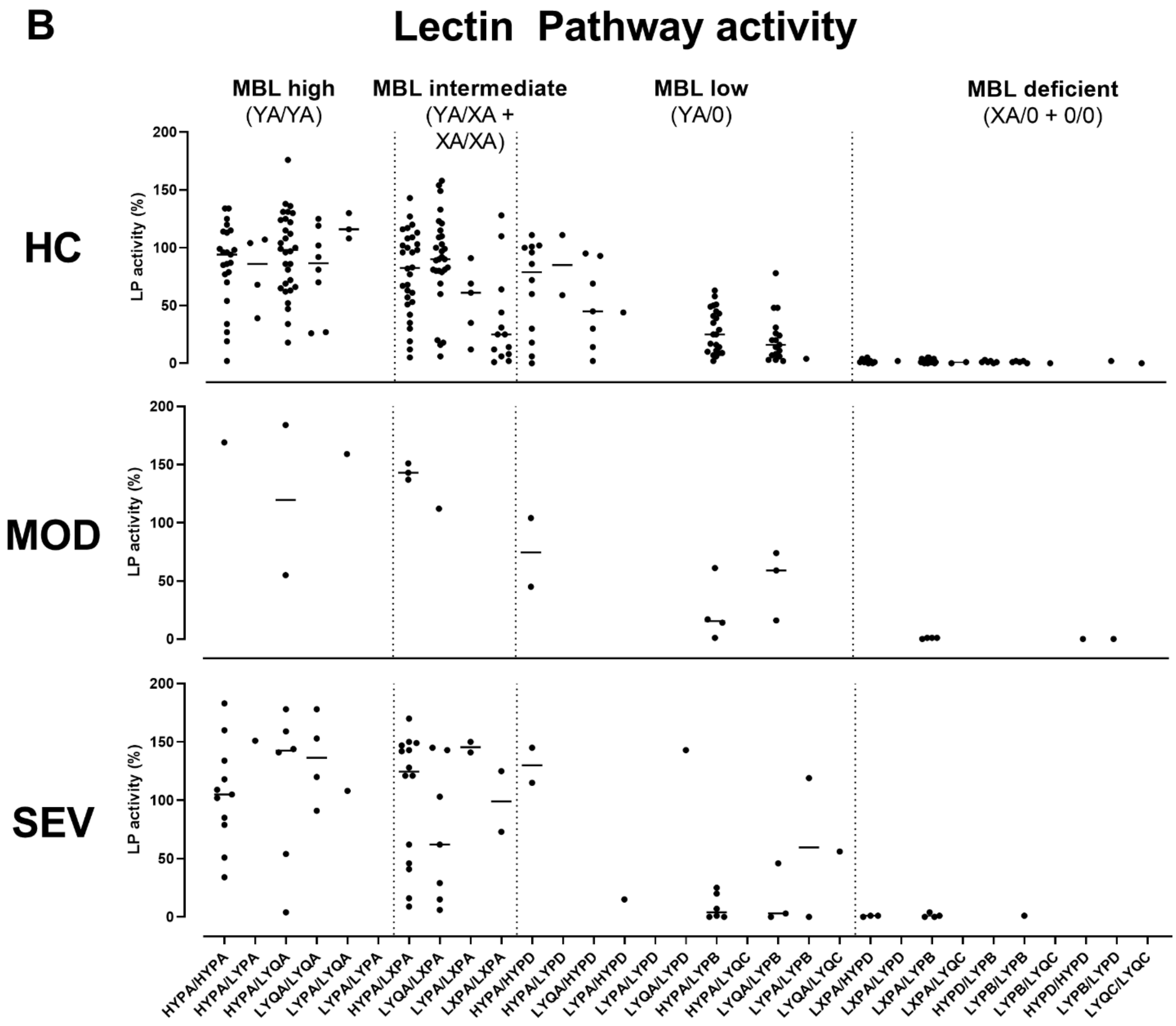

Supplementary Figure 4: MBL levels (CAM) and Lectin pathway activity (BUD) in healthy controls and COVID-19 patients, stratified according to *MBL2* genotype groups and disease severity. Short *MBL2* haplotype combinations were merged into four different groups: 1) MBL high (YA/YA), 2) MBL intermediate (YA/XA+XA/XA), 3) MBL low (YA/0 (including YA/YB, YA/YC and YA/YD)), and 4) MBL deficient (XA/0+0/0 (including 0/0 for allele A, XA/YB, XA/YC and XA/YD)). After genetic grouping, groups were furthermore stratified according to disease severity (HC, MOD, SEV), and MBL (A) as well as Lectin pathway activity levels (B) of individuals are presented.

Abbreviations: HC, Healthy controls; MOD, moderate patients group; SEV, severe patients group; LP, lectin pathway.

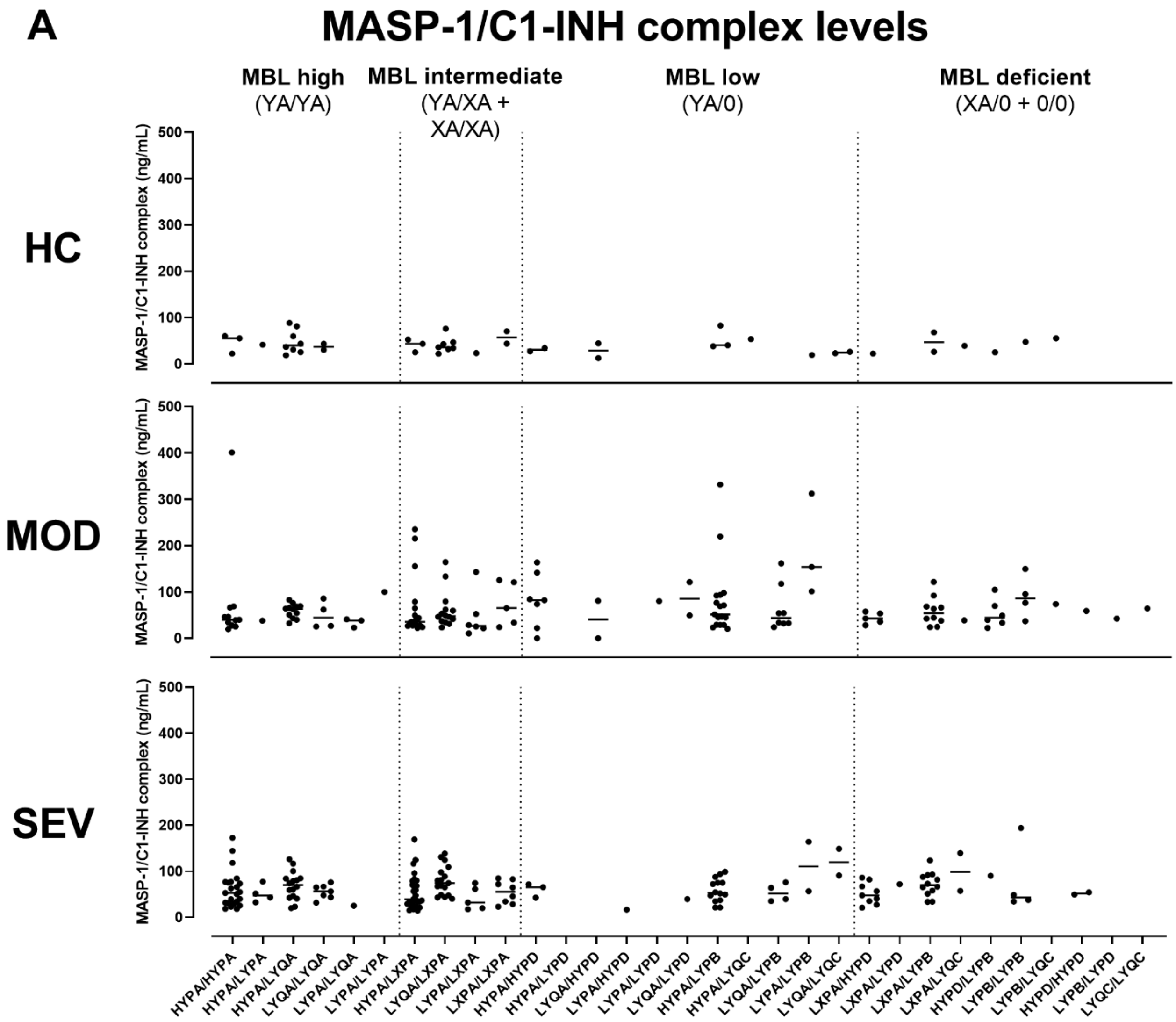
A
MASP-1/C1-INH complex levels
MBL high
(YA/YA)
MBL intermediate
(YA/XA +
XA/XA)
MBL low
(YA/0)
MBL deficient
(XA/0 + 0/0)
HC
MOD
SEV
MASP-1/C1-INH complex (ng/mL)
0
100
200
300
400
500
HYPA/HYPA
HYPA/LYPA
HYPA/LYQA
LYQA/LYQA
LYPA/LYQA
LYPA/LYPA
HYPA/LXPA
LYQA/LXPA
LYPA/LXPA
LXPA/LXPA
HYPA/HYPD
HYPA/LYPD
LYQA/HYPD
LYPA/HYPD
LYPA/LYPD
LYQA/LYPD
HYPA/LYPB
HYPA/LYQC
LYQA/LYPB
LYPA/LYPB
LYQA/LYQC
LXPA/HYPD
LXPA/LYPD
LXPA/LYPB
LXPA/LYQC
HYPD/LYPB
LYPB/LYPB
LYPB/LYQC
HYPD/HYPD
LYPB/LYPD
LYQC/LYQC

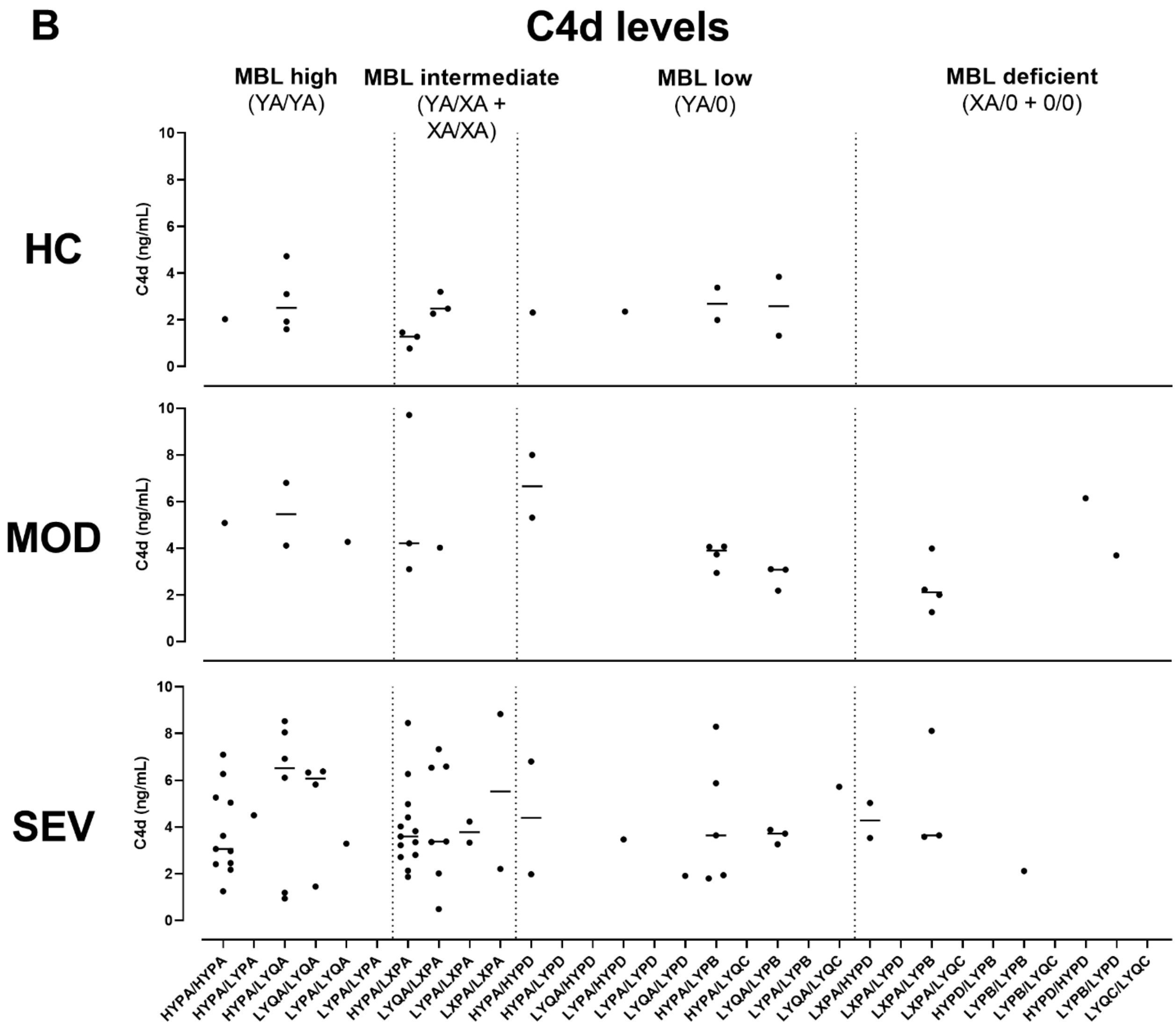

Supplementary Figure 5: MASP-1/C1-INH complex and C4d levels in healthy controls and COVID-19 patients, stratified according to *MBL2* genotype groups and disease severity. Short *MBL2* haplotype combinations were merged into four different groups: 1) MBL high (YA/YA), 2) MBL intermediate (YA/XA+XA/XA), 3) MBL low (YA/0 (including YA/YB, YA/YC and YA/YD)), and 4) MBL deficient (XA/0+0/0 (including 0/0 for allele A, XA/YB, XA/YC and XA/YD)). After genetic grouping, groups were furthermore stratified according to disease severity (HC, MOD, SEV), and MASP-1/C1-INH complex (A) as well as C4d levels (B) of individuals are presented.
Abbreviations: HC, Healthy controls; MOD, moderate patients group; SEV, severe patients group.

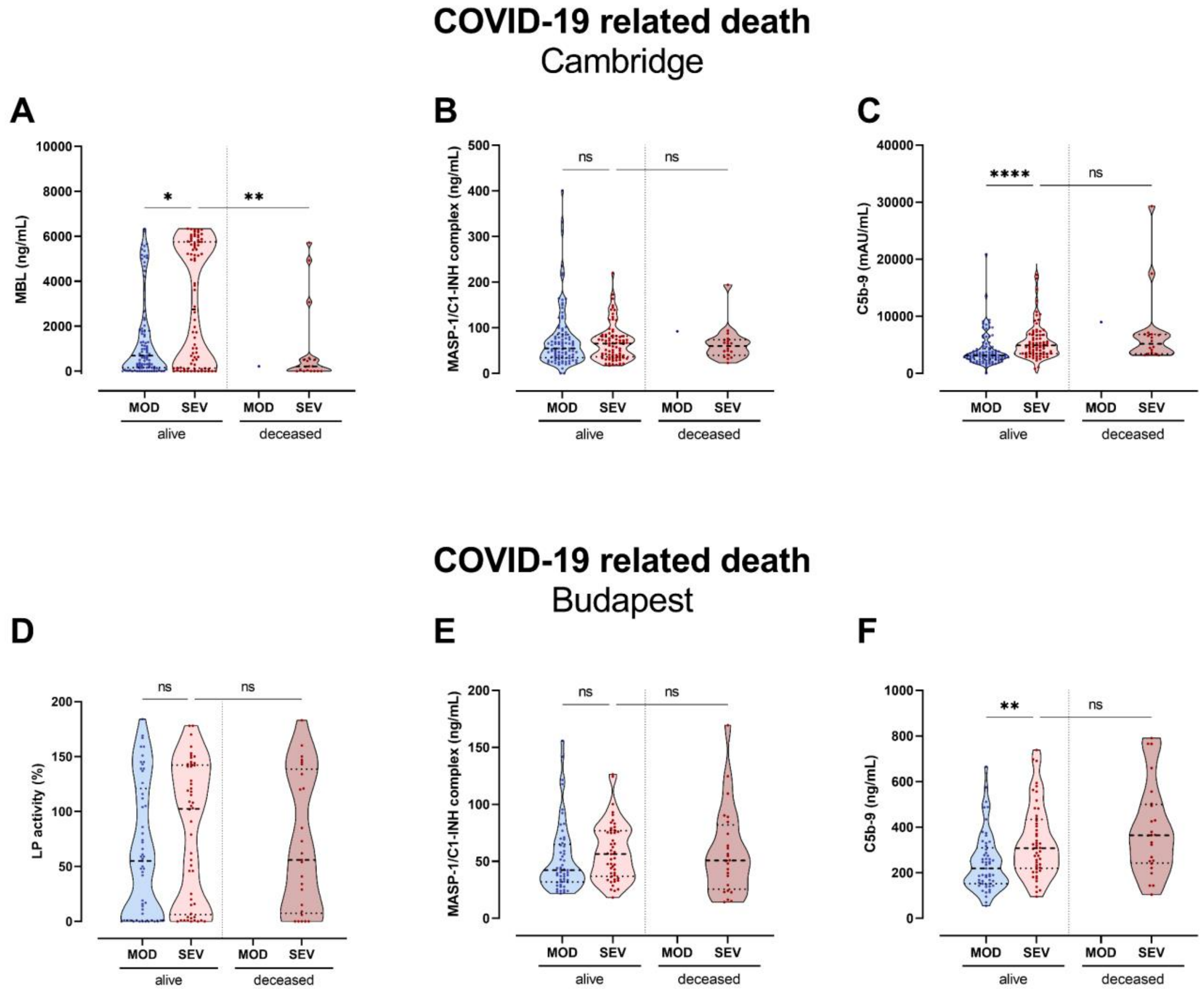

Supplementary Figure 6: Relationship between complement markers and mortality in moderate (MOD) and severe (SEV) COVID-19 patients. Levels of complement markers MBL (A), MASP-1/C1-INH complex (B) and TCC/C5b-9 (C) in the Cambridge cohort and Lectin pathway activity (D), MASP-1/C1-INH complex (E) and TCC/C5b-9 (F) of the Budapest cohort were stratified according to survival (alive vs. deceased) and disease severity (MOD vs. SEV). Differences between associated groups were analysed using the Kruskal-Wallis test with Dunn's multiple comparison post-hoc test, while asterisks indicate significant differences (*p<0.05, **p<0.01, ****p<0.0001). Non-significant differences are indicated (ns).

Abbreviations: MASP-1/C1-INH, MASP-1/C1-INH complex; LP, lectin pathway activity; MOD, moderate patients group; SEV, severe patients group.

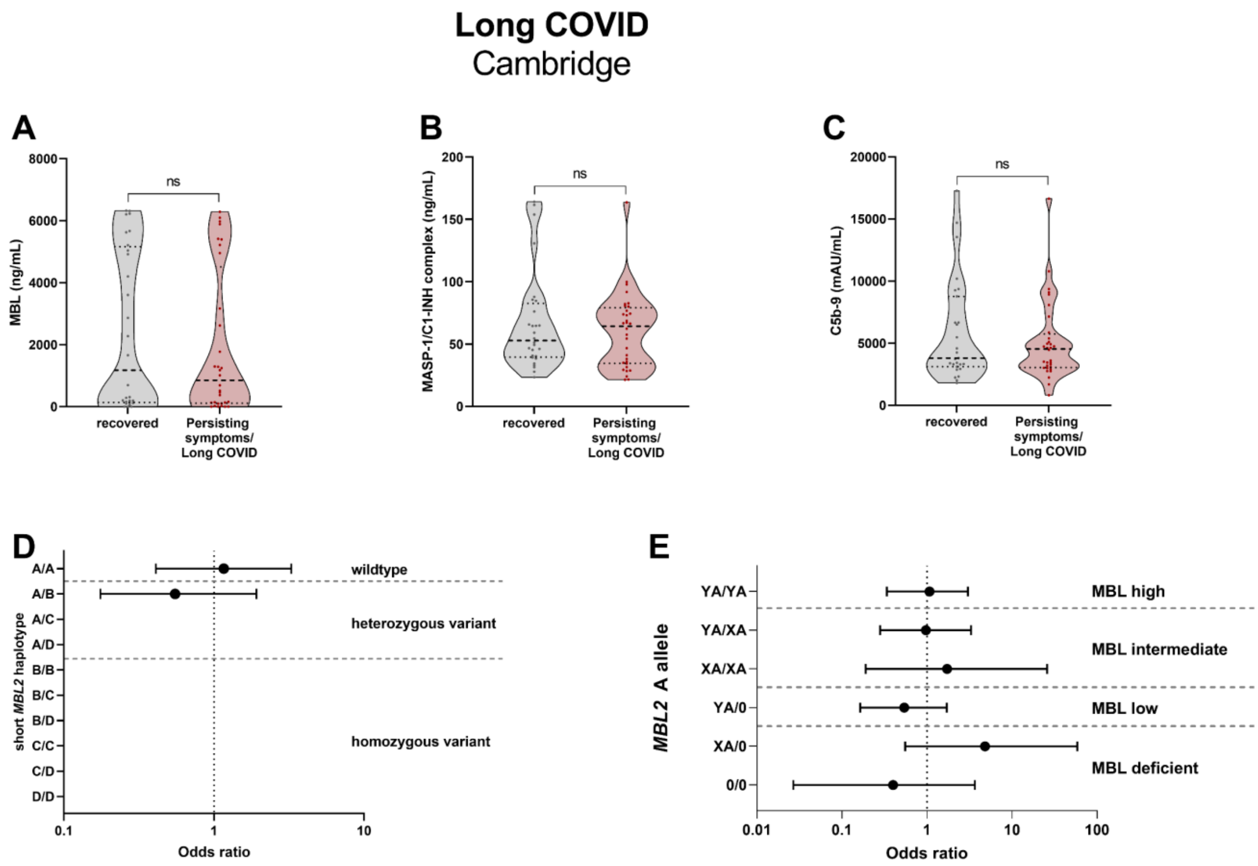

Supplementary Figure 7: Relationship between complement markers, MBL genotype and Long COVID in COVID-19 patients. Levels of complement markers MBL (A), MASP-1/C1-INH complex (B) and TCC/C5b-9 (C) in the Cambridge cohort were stratified according to their Long COVID status (recovered vs. persisting symptoms/Long COVID). Differences between the two groups were analysed using the Mann-Whitney test, while non-significant differencesare indicated (ns).

Forest plot displaying Odds ratios (ORs) with 95% confidence intervals (CIs) for the development of Long COVID, stratified according to the *MBL2* A carrier state (D) as well as according to the *MBL2* genotype groups (E). Odds ratios of 0 or infinite are not indicated on the forest plots. p-values are presented as non-corrected for multiple testing. Threshold for significance taking into account multiple testing are p=0.0050 for the *MBL2* A allele, and p=0.0083 for the short *MBL2* haplotype (Benjamini-Hochberg correction).

Abbreviations: MASP-1/C1-INH, MASP-1/C1-INH complex; C5b-9, terminal complement complex.

2 Supplementary Tables

Supplementary Table 1: Frequencies of short *MBL2* haplotype combinations in healthy controls and COVID-19 cases of the Cambridge cohort (CAM).

	**cases (n=46)**		**controls (n=192)**		**Odds ratio (95% Confidence interval)**	**p-value[a]**
	n	%	n	%		
**YA/YA**	50	26.0%	14	30.4%	0.805 (0.412-1.668)	0.5802
**YA/XA**	52	27.1%	11	23.9%	1.182 (0.574-2.582)	0.7140
**YA/0**	39	20.3%	12	26.1%	0.722 (0.347-1.519)	0.4249
**XA/XA**	11	5.7%	2	4.3%	1.337 (0.304-6.221)	>0.9999
**XA/0**	27	14.1%	4	8.7%	1.718 (0.592-4.764)	0.4653
**0/0**	13	6.8%	3	6.5%	1.041 (0.319-3.555)	>0.9999

[a] p-values are presented as non-corrected for multiple testing. Threshold for significance taking into account multiple testing is p=0.0083 (Benjamini-Hochberg correction).

Supplementary Table 2: Frequencies of short *MBL2* haplotype combinations in healthy controls and COVID-19 cases of the Budapest cohort (BUD).

	**cases (n=123)**		**controls (n=331)**		**Odds ratio (95% Confidence interval)**	**p-value[a]**
	n	%	n	%		
**YA/YA**	33	26.8%	88	26.6%	1.013 (0.642-1.608)	>0.9999
**YA/XA**	35	28.5%	82	24.8%	1.208 (0.751-1.913)	0.4690
**YA/0**	31	25.2%	85	25.7%	0.975 (0.608-1.571)	>0.9999
**XA/XA**	2	1.6%	15	4.5%	0.348 (0.078-1.467)	0.1752
**XA/0**	14	11.4%	40	12.1%	0.934 (0.498-1.770)	>0.9999
**0/0**	8	6.5%	21	6.3%	1.027 (0.471-2.318)	>0.9999

[a] p-values are presented as non-corrected for multiple testing. Threshold for significance taking into account multiple testing is p=0.0083 (Benjamini-Hochberg correction).

Supplementary Table 3: Distribution of exonic wildtype (A) and variant alleles (B, C, and D) of *MBL2* SNPs in healthy controls and COVID-19 cases of the Cambridge cohort (CAM)

	**controls (n=46)**		**cases (n=192)**		**Odds ratio (95% Confidence interval)**	**p-value[a]**
	n	%	n	%		
**Wildtype**						
**A/A**	**27**	**58.7%**	**113**	**58.9%**	**1.007 (0.527-1.937)**	**>0.9999**
**Heterozygous**						
**A/B**	7	15.2%	43	22.4%	1.608 (0.703-4.088)	0.3210
**A/C**	4	8.7%	4	2.1%	0.223 (0.064-0.800)	0.0473
**A/D**	5	10.9%	19	9.9%	0.901 (0.317-2.315)	0.7894
**Total A/0**	**16**	**34.8%**	**66**	**34.4%**	**0.982 (0.498-1.871)**	**>0.9999**
**Homozygous variant**						
**B/B**	1	2.2%	4	2.1%	0.957 (0.153-11.950)	>0.9999
**B/C**	1	2.2%	1	0.5%	0.236 (0.012-4.570)	0.3499
**B/D**	1	2.2%	5	2.6%	1.203 (0.158-14.470)	>0.9999
**C/C**	0	0.0%	1	0.5%	infinity (0.027-infinity)	>0.9999
**C/D**	0	0.0%	0	0.0%	-	-
**D/D**	0	0.0%	2	1.0%	infinity (0.110-infinity)	>0.9999
**Total 0/0**	**3**	**6.5%**	**13**	**6.8%**	**1.041 (0.319-3.555)**	**>0.9999**

[a] p-values are presented as non-corrected for multiple testing. Threshold for significance taking into account multiple testing is p=0.0045 (Benjamini-Hochberg correction).

Supplementary Table 4: Distribution of exonic wildtype (A) and variant alleles (B, C, and D) of *MBL2* SNPs in healthy controls and COVID-19 cases of the Budapest cohort (BUD)

	**controls (n=331)**		**cases (n=123)**		**Odds ratio (95% Confidence interval)**	**p-value[a]**
	n	%	n	%		
**Wildtype**						
**A/A**	**185**	**55.9%**	**70**	**56.9%**	**1.042 (0.680-1.581)**	**0.9153**
**Heterozygous**						
**A/B**	79	23.9%	30	24.4%	1.029 (0.631-1.680)	0.9022
**A/C**	3	0.9%	1	0.8%	0.896 (0.068-6.056)	>0.9999
**A/D**	43	13.0%	14	11.4%	0.860 (0.462-1.608)	0.7506
**Total A/0**	**125**	**37.8%**	**45**	**36.6%**	**0.951 (0.690-1.602)**	**0.828**
**Homozygous variant**						
**B/B**	8	2.4%	4	3.3%	1.357 (0.447-4.480)	0.7422
**B/C**	1	0.3%	0	0.0%	0.000 (0.000-24.220)	>0.9999
**B/D**	8	2.4%	3	2.4%	1.009 (0.286-3.339)	>0.9999
**C/C**	1	0.3%	0	0.0%	0.000 (0.0000-24.220)	>0.9999
**C/D**	1	0.3%	0	0.0%	0.000 (0.0000-24.220)	>0.9999
**D/D**	2	0.6%	1	0.8%	1.348 (0.092-11.670)	>0.9999
**Total 0/0**	**21**	**6.3%**	**8**	**6.5%**	**1.027 (0.471-2.318)**	**>0.9999**

[a] p-values are presented as non-corrected for multiple testing. Threshold for significance taking into account multiple testing is p=0.0042 (Benjamini-Hochberg correction).

Supplementary Table 5: Distribution of long *MBL2* haplotype combinations in controls vs. cases. Frequencies in controls and cases were compared using fisher's exact test. Results are given in odds ratios (OR) and 95% confidence intervals (95% CI), together with their respective p-value.

	controls (n=373)		cases (n=314)		Odds ratio (95% Confidence interval)	p-value[a]
	n	%	n	%		
**HYPA/LXPA**	41	11.0%	45	14.3%	1.355 (0.859-2.153)	0.204
**LXPA/LYQA**	44	11.8%	31	9.9%	0.819 (0.504-1.327)	0.462
**HYPA/LYQA**	46	12.3%	28	8.9%	0.696 (0.429-1.141)	0.174
**HYPA/LYPB**	36	9.7%	32	10.2%	1.062 (0.640-1.740)	0.898
**HYPA/HYPA**	32	8.6%	34	10.8%	1.294 (0.777-2.169)	0.363
**LXPA/LYPB**	26	7.0%	23	7.3%	1.055 (0.579-1.882)	0.883
**LYQA/LYPB**	22	5.9%	12	3.8%	0.634 (0.318-1.275)	0.223
**LXPA/LXPA**	17	4.6%	13	4.1%	0.904 (0.448-1.833)	0.853
**HYPA/HYPD**	17	4.6%	10	3.2%	0.689 (0.324-1.512)	0.432
**LXPA/HYPD**	12	3.2%	14	4.5%	1.404 (0.669-3.082)	0.427
**LYQA/LYQA**	12	3.2%	11	3.5%	1.092 (0.496-2.559)	0.835
**LXPA/LYPA**	8	2.1%	11	3.5%	1.656 (0.682-4.019)	0.352
**LYPB/LYPB**	9	2.4%	8	2.5%	1.057 (0.413-2.590)	>0.999
**HYPD/LYPB**	8	2.1%	7	2.2%	1.037 (0.394-2.876)	>0.999
**LYQA/HYPD**	13	3.5%	2	0.6%	0.178 (0.040-0.678)	0.016
**HYPA/LYPA**	6	1.6%	5	1.6%	0.990 (0.343-3.361)	>0.999
**LYPA/LYPB**	2	0.5%	6	1.9%	3.614 (0.869-17.700)	0.151
**LYPA/LYQA**	3	0.8%	4	1.3%	1.591 (0.424-6.352)	0.708
**LXPA/LYQC**	4	1.1%	3	1.0%	0.890 (0.223-3.337)	>0.999
**HYPD/HYPD**	2	0.5%	3	1.0%	1.789 (0.363-10.130)	0.665
**LYQA/LYQC**	2	0.5%	2	0.6%	1.189 (0.185-7.627)	>0.999
**LYQA/LYPD**	0	0.0%	3	1.0%	Infinity (1.032-infinitiy)	0.095
**LYPB/LYQC**	2	0.5%	1	0.3%	0.593 (0.041-5.121)	>0.999
**HYPD/LYPA**	1	0.3%	1	0.3%	1.188 (0.062-22.640)	>0.999
**LXPA/LYPD**	1	0.3%	1	0.3%	1.188 (0.062-22.640)	>0.999
**LYPA/LYPD**	1	0.3%	1	0.3%	1.188 (0.062-22.640)	>0.999
**LYPB/LYPD**	1	0.3%	1	0.3%	1.188 (0.062-22.640)	>0.999
**LYQC/LYQC**	1	0.3%	1	0.3%	1.188 (0.062-22.640)	>0.999
**HYPA/LYPD**	2	0.5%	0	0.0%	0.000 (0.000-2.567)	0.503
**LYPA/LYPA**	0	0.0%	1	0.3%	Infinity (0.132-infinity)	0.457
**HYPA/LYQC**	1	0.3%	0	0.0%	0.000 (0.000-10.690)	>0.999
**HYPD/LYQC**	1	0.3%	0	0.0%	0.000 (0.000-10.690)	>0.999

[a] p-values are presented as non-corrected for multiple testing. Threshold for significance taking into account multiple testing is p=0.0016 (Benjamini-Hochberg correction).

Supplementary Table 6: Distribution of long *MBL2* haplotype combinations in moderate vs. severe cases. Frequencies in moderate and severe COVID-19 cases were compared using fisher's exact test. Results are given in odds ratios (OR) and 95% confidence intervals (95% CI), together with their respective p-value. Abbreviations: n, number; OR, odds ratio; CI, confidence interval.

	**moderate (n=144)**		**severe (n=170)**		**Odds ratio (95% Confidence interval)**	**p-value[a]**
	n	%	n	%		
**HYPA/LXPA**	17	11.8%	28	16.5%	1.473 (0.783-2.842)	0.261
**LXPA/LYQA**	14	9.7%	17	10.0%	1.032 (0.499-2.253)	>0.999
**HYPA/LYQA**	12	8.3%	16	9.4%	1.143 (0.515-2.521)	0.843
**HYPA/LYPB**	17	11.8%	15	8.8%	0.723 (0.345-1.470)	0.455
**HYPA/HYPA**	11	7.6%	23	13.5%	1.892 (0.888-4.156)	0.104
**LXPA/LYPB**	10	6.9%	13	7.6%	1.110 (0.468-2.584)	0.832
**LYQA/LYPB**	8	5.6%	4	2.4%	0.410 (0.135-1.337)	0.154
**LXPA/LXPA**	5	3.5%	8	4.7%	1.373 (0.425-3.796)	0.778
**HYPA/HYPD**	7	4.9%	3	1.8%	0.352 (0.098-1.292)	0.195
**LXPA/HYPD**	5	3.5%	9	5.3%	1.554 (0.530-4.219)	0.586
**LYQA/LYQA**	4	2.8%	7	4.1%	1.503 (0.469-4.665)	0.557
**LXPA/LYPA**	6	4.2%	5	2.9%	0.697 (0.237-2.421)	0.760
**LYPB/LYPB**	4	2.8%	4	2.4%	0.843 (0.242-2.943)	>0.999
**HYPD/LYPB**	6	4.2%	1	0.6%	0.136 (0.012-0.844)	0.051
**LYQA/HYPD**	2	1.4%	0	0.0%	0.000 (0.000-1.827)	0.210
**HYPA/LYPA**	1	0.7%	4	2.4%	3.446 (0.562-42.430)	0.380
**LYPA/LYPB**	3	2.1%	3	1.8%	0.844 (0.195-3.663)	>0.999
**LYPA/LYQA**	3	2.1%	1	0.6%	0.278 (0.021-1.889)	0.336
**LXPA/LYQC**	1	0.7%	2	1.2%	1.702 (0.196-24.830)	>0.999
**HYPD/HYPD**	1	0.7%	2	1.2%	1.702 (0.196-24.830)	>0.999
**LYQA/LYQC**	0	0.0%	2	1.2%	infinity (0.392-infinity)	0.502
**LYQA/LYPD**	2	1.4%	1	0.6%	0.420 (0.028-3.653)	0.595
**LYPB/LYQC**	1	0.7%	0	0.0%	0.000 (0.000-7.624)	0.459
**HYPD/LYPA**	0	0.0%	1	0.6%	infinity (0.094-infinity)	>0.999
**LXPA/LYPD**	0	0.0%	1	0.6%	infinity (0.094-infinity)	>0.999
**LYPA/LYPD**	1	0.7%	0	0.0%	0.000 (0.000-7.624)	0.459
**LYPB/LYPD**	1	0.7%	0	0.0%	0.000 (0.000-7.624)	0.459
**LYQC/LYQC**	1	0.7%	0	0.0%	0.000 (0.000-7.624)	0.459
**HYPA/LYPD**	0	0.0%	0	0.0%	-	-
**LYPA/LYPA**	1	0.7%	0	0.0%	0.000 (0.000-7.624)	0.459
**HYPA/LYQC**	0	0.0%	0	0.0%	-	-
**HYPD/LYQC**	0	0.0%	0	0.0%	-	-

[a] p-values are presented as non-corrected for multiple testing. Threshold for significance taking into account multiple testing is p=0.0017 (Benjamini-Hochberg correction).

Supplementary Table 7: Allele frequencies of *MBL2* alleles in controls and COVID-19 cases. Allele frequencies in controls and COVID-19 cases were compared using fisher's exact test. Results are given in odds ratios (OR) and 95% confidence intervals, together with their respective p-value.

	**controls (n=754)**		**cases (n=628)**		**Odds ratio (95% Confidence interval)**	**p-value[a]**
	n	%	n	%		
**A**	565	74.9%	477	75.7%	1.043 (0.819-1.333)	0.755
**B**	115	15.3%	98	15.6%	1.024 (0.763-1.367)	0.881
**C**	12	1.6%	8	1.3%	0.795 (0.338-1.886)	0.658
**D**	62	8.2%	47	7.5%	0.900 (0.603-1.343)	0.618
**Y**	583	77.3%	474	75.5%	0.903 (0.704-1.158)	0.445
**X**	171	22.7%	154	24.5%	1.108 (0.861-1.423)	0.445
**H**	269	36.1%	228	36.3%	1.011 (0.809-1.262)	0.955
**L**	477	63.9%	400	63.7%	0.989 (0.793-1.237)	0.955
**P**	580	77.7%	516	82.2%	1.319 (1.010-1.720)	0.043
**Q**	166	22.3%	112	17.8%	0.758 (0.581-0.990)	0.043

[a] p-values are presented as non-corrected for multiple testing. Threshold for significance taking into account multiple testing is p=0.0050 (Benjamini-Hochberg correction).

Supplementary Table 8: Distribution of exonic wildtype (A) and variant alleles (B, C, and D) of *MBL2* SNPs in COVID-19 cases of the Cambridge cohort (CAM) and the Budapest cohort (BUD), stratified according to disease severity (moderate (MOD) and severe (SEV)) and COVID-19 related mortality as an outcome.

	COVID-19 related mortality - A allele					
		CAM			BUD	
		MOD	SEV		MOD	SEV
A/A	alive	52	52	alive	22	31
	deceased	0	6	deceased	0	17
non-A/A	alive	41	24	alive	27	19
	deceased	1	11	deceased	0	7
A/B	alive	21	14	alive	15	12
	deceased	1	5	deceased	0	3
non-A/B	alive	72	62	alive	34	38
	deceased	0	12	deceased	0	21
A/C	alive	1	3	alive	0	1
	deceased	0	0	deceased	0	0
non-A/C	alive	92	73	alive	49	49
	deceased	1	17	deceased	0	24
A/D	alive	11	5	alive	6	5
	deceased	0	3	deceased	0	3
non-A/D	alive	82	71	alive	43	45
	deceased	1	14	deceased	0	21
B/B	alive	1	1	alive	3	1
	deceased	0	2	deceased	0	0
non-B/B	alive	92	75	alive	46	49
	deceased	1	15	deceased	0	24
B/C	alive	1	0	alive	0	0
	deceased	0	0	deceased	0	0
non-B/C	alive	92	76	alive	49	50
	deceased	1	17	deceased	0	24
B/D	alive	5	0	alive	2	0
	deceased	0	0	deceased	0	1
non-B/D	alive	88	76	alive	47	50
	deceased	1	17	deceased	0	23
C/C	alive	1	0	alive	0	0
	deceased	0	0	deceased	0	0
non-C/C	alive	92	76	alive	49	50
	deceased	1	17	deceased	0	24
D/D	alive	0	1	alive	1	0
	deceased	0	1	deceased	0	0
non-D/D	alive	93	75	alive	48	50
	deceased	1	16	deceased	0	24
	**sum alive**	**93**	**76**	**sum alive**	**49**	**50**
	**sum deceased**	**1**	**17**	**sum deceased**	**0**	**24**

Supplementary Table 9: Frequencies of short *MBL2* haplotype combinations in COVID-19 cases of the Cambridge cohort (CAM) and the Budapest cohort (BUD), stratified according to disease severity (moderate (MOD) and severe (SEV)) and COVID-related mortality as an outcome.

	**COVID-19 related mortality - short haplotypes**					
		**CAM**			**BUD**	
		**MOD**	**SEV**		**MOD**	**SEV**
YA/YA	alive	22	25	alive	10	16
	deceased	0	2	deceased	0	7
non-YA/YA	alive	71	51	alive	39	34
	deceased	1	15	deceased	0	17
YA/XA	alive	25	21	alive	12	14
	deceased	0	4	deceased	0	9
non-YA/XA	alive	68	55	alive	37	36
	deceased	1	13	deceased	0	15
XA/XA	alive	5	6	alive	0	1
	deceased	0	0	deceased	0	1
non-XA/XA	alive	88	70	alive	49	49
	deceased	1	17	deceased	0	23
YA/0	alive	25	8	alive	14	11
	deceased	1	4	deceased	0	6
non-YA/0	alive	68	68	alive	35	39
	deceased	0	13	deceased	0	18
XA/0	alive	8	14	alive	7	7
	deceased	0	4	deceased	0	0
non-XA/0	alive	85	62	alive	42	43
	deceased	1	13	deceased	0	24
0/0	alive	8	2	alive	6	1
	deceased	0	3	deceased	0	1
non-0/0	alive	85	74	alive	43	49
	deceased	1	14	deceased	0	23
	**sum alive**	**93**	**76**		**49**	**50**
	**sum deceased**	**1**	**17**		**0**	**24**

Supplementary Table 10: Distribution of exonic wildtype (A) and variant alleles (B, C, and D) of *MBL2* SNPs in COVID-19 cases of the Cambridge cohort (CAM), stratified according to disease severity (moderate (MOD) and severe (SEV)) and the development of Long COVID (LC) as an outcome.

		Long COVID - A allele	
		CAM	
		MOD	SEV
A/A	LC	7	12
	no LC	6	9
non-A/A	LC	7	5
	no LC	8	3
A/B	LC	4	3
	no LC	7	2
non-A/B	LC	10	14
	no LC	7	10
A/C	LC	0	1
	no LC	0	0
non-A/C	LC	14	16
	no LC	14	12
A/D	LC	2	1
	no LC	0	0
non-A/D	LC	12	16
	no LC	14	12
B/B	LC	0	0
	no LC	0	0
non-B/B	LC	14	17
	no LC	14	12
B/C	LC	1	0
	no LC	0	0
non-B/C	LC	13	17
	no LC	14	12
B/D	LC	0	0
	no LC	0	0
non-B/D	LC	14	17
	no LC	14	12
C/C	LC	0	0
	no LC	1	0
non-C/C	LC	14	17
	no LC	13	12
D/D	LC	0	0
	no LC	0	1
non-D/D	LC	14	17
	no LC	14	11
	**sum LC**	**14**	**17**
	**sum no LC**	**14**	**12**

Supplementary Table 11: Frequencies of short *MBL2* haplotype combinations in COVID-19 cases of the Cambridge cohort (CAM), stratified according to disease severity (moderate (MOD) and severe (SEV)) and the development of Long COVID (LC) as an outcome.

	**Long COVID - short haplotypes**		
		**CAM**	
		**MOD**	**SEV**
YA/YA	no LC	4	4
	LC	4	6
non-YA/YA	no LC	10	8
	LC	10	11
YA/XA	no LC	2	4
	LC	3	4
non-YA/XA	no LC	12	8
	LC	11	13
XA/XA	no LC	0	1
	LC	0	2
non-XA/XA	no LC	14	11
	LC	14	15
YA/0	no LC	7	1
	LC	4	2
non-YA/0	no LC	7	11
	LC	10	15
XA/0	no LC	0	1
	LC	2	3
non-XA/0	no LC	14	11
	LC	12	14
0/0	no LC	1	1
	LC	1	0
non-0/0	no LC	13	11
	LC	13	17
	**sum LC**	**14**	**12**
	**sum no LC**	**14**	**17**
